# Humanized In Vivo Bone Tissue Engineering: In Vitro Preculture Conditions Control the Structural, Cellular, and Matrix Composition of Humanized Bone Organs

**DOI:** 10.1002/adhm.202401939

**Published:** 2024-10-23

**Authors:** Agathe Bessot, Flavia Medeiros Savi, Jennifer Gunter, Jayanti Mendhi, Shahrouz Amini, David Waugh, Jacqui McGovern, Dietmar W. Hutmacher, Nathalie Bock

**Affiliations:** ^1^ School of Biomedical Sciences Faculty of Health and Translational Research Institute (TRI) Queensland University of Technology (QUT) Brisbane QLD 4102 Australia; ^2^ Centre for Biomedical Technologies QUT Brisbane QLD 4000 Australia; ^3^ Max Planck Queensland Centre Brisbane QLD 4000 Australia; ^4^ Australian Research Council (ARC) Training Centre for Multiscale 3D Imaging Modelling and Manufacturing (M3D Innovation) Queensland University of Technology Brisbane QLD 4000 Australia; ^5^ Australian Prostate Cancer Research Centre (APCRC‐Q) QUT Brisbane QLD 4102 Australia; ^6^ Central Analytical Research Facility QUT Brisbane QLD 4102 Australia; ^7^ Department of Biomaterials Max Planck Institute of Colloids and Interfaces 14476 Potsdam Germany; ^8^ Centre for Cancer Biology University of South Australia Adelaide South Australia Australia; ^9^ Australian Research Council (ARC) Training Centre for Cell and Tissue Engineering Technologies (CTET) QUT Brisbane QLD 4000 Australia

**Keywords:** bone structure, bone tissue engineering (BTE), extracellular matrix, humanized mouse model, in vivo bone model, mineralization, osteogenic differentiation (OD)

## Abstract

Bone tissue engineering (BTE) has long sought to elucidate the key factors controlling human/humanized bone formation for regenerative medicine and disease modeling applications, yet with no definitive answers due to the high number and co‐dependency of parameters. This study aims to clarify the relative impacts of in vitro biomimetic ‘preculture composition’ and ‘preculture duration’ before in vivo implantation as key criteria for the optimization of BTE design. These parameters are directly related to in vitro osteogenic differentiation (OD) and mineralization and are being investigated across different osteoprogenitor‐loaded biomaterials, specifically fibrous calcium phosphate‐polycaprolactone (CaP‐mPCL) scaffolds and gelatin methacryloyl (GelMA) hydrogels. The results show that OD and mineralization levels prior to implantation, enhanced by a mineralization medium supplement to the osteogenic medium (OM), significantly improve ectopic BTE outcomes, regardless of the biomaterial type. Specifically, preculture conditions are pivotal in achieving more faithful mimicry of human bone structure, cellular and extracellular matrix composition and organization, and provide control over bone marrow composition. This work emphasizes the potential of using biomimetic culture compositions, specifically the addition of a mineralization medium as a cost‐effective and straightforward approach to enhance BTE outcomes, facilitating rapid development of bone models with superior quality and resemblance to native bone.

## Graphical abstract

Mineralization prior to in vivo implantation is shown to control the outcomes of humanized bone organs. Modulated by preculture duration and culture composition as critical parameters, the findings demonstrate that advanced biomaterials loaded with osteogenic cells and minerals exhibiting higher mineralization levels yield to superior humanized ectopic bone organs, with strict control over ossification, cellular/extracellular maturation, and structural bone‐like organization.

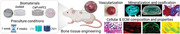

## 1 Introduction

Ectopic bone tissue engineering (BTE) in subcutaneous animal models has emerged as a pivotal technique for exploring bone biology and pathophysiology.^[^ ^1^ ^]^ Ectopic BTE offers more controlled formation of humanized bone without interference from host bone cells, unlike orthotopic models (in the host bone). A critical challenge in BTE however lies in striking an effective balance between achieving a human bone organ with faithful extracellular matrix (ECM) recapitulation and the practical limitations of current methodologies.^[^ ^2^ ^]^ Recent advancements in biomaterials and tissue engineering have led to nuanced control over the ECM's chemical, biological, and mechanical attributes.^[^ ^3^ ^]^ Notably, advanced functionalized and composite biomaterials have been developed to achieve more relevant bone‐like structures,^[^ ^4^ ^]^ allowing for enhanced control of the mechanical properties, mineralization levels, and the resemblance to natural bone ECM.^[^ ^5^ ^]^

Mineral ECM‐mimicry involves replicating the inorganic component of bone, which is primarily composed of calcium and phosphate minerals (CaP).^[^ ^6^, ^7^, ^8^ ^]^ Biodegradable medical‐grade polycaprolactone (mPCL) scaffolds coated with calcium and phosphate (CaP‐mPCL) have been widely used and characterized for instance for BTE in ectopic^[^ ^9^, ^10^, ^11^ ^]^ and orthotopic animal models.^[^ ^12^, ^13^ ^]^ CaP‐mPCL scaffolds not only provide structural support and improved bioactivity, but they also enhance osteoinductivity, leading to higher mineralization levels compared to uncoated scaffolds.^[^ ^8^ ^]^ Conversely, protein ECM‐mimicry aims to replicate the organic component of the bone ECM, notably including collagen.^[^ ^6^, ^14^ ^]^ Gelatin methacryloyl (GelMA) hydrogels, derived from collagen, offer a semi‐synthetic matrix with tunable mechanical properties, faster degradability, and mimic the natural ECM protein environment, promoting osteogenic differentiation (OD)^[^ ^15^ ^]^ and supporting ectopic bone formation.^[^ ^16^, ^17^, ^18^, ^19^ ^]^ Although protein and mineral ECM‐mimicry approaches are fundamentally different, both have shown significant success in bone formation and regeneration.^[^ ^9^, ^10^, ^11^, ^16^, ^17^, ^18^, ^19^ ^]^ However, the specific mechanisms enabling successful BTE‐driven human bone formation are still not fully understood. The variety in existing protocols makes it difficult to identify the key parameters, and their relative importance, to achieve satisfactory control over BTE‐driven bone mimicry.

In BTE, an established factor considered as a key contributor to engineered humanized bone is the use of primary human osteoprogenitors, enhancing biofunctionalization^[^ ^20^ ^]^ and leading to superior bone tissue ossification when pre‐differentiated into osteoblasts.^[^ ^8^, ^21^ ^]^ However, to achieve satisfactory osteoblastic differentiation and ECM deposition, a variety of options related to in vitro biomimetic culture compositions and durations prior to implantation are available. Osteogenic medium (OM), containing supplements driving osteogenesis (dexamethasone, ascorbic acid, β‐glycerophosphate), is a common approach in in vitro OD,^[^ ^8^, ^17^, ^22^ ^]^ but alternative strategies, such as supplementing the culture medium with growth factors (BMPs)^[^ ^23^ ^]^ or nanoparticles (HA, chitosan)^[^ ^24^ ^]^ have emerged as promising techniques for enhanced cellular differentiation and mineral deposition. While effective, these alternative approaches are costly and not always reproducible. The use of a culture medium enriched with calcium and phosphorus ions has recently been proposed as a superior and more affordable solution with comparable efficacy on osteogenesis and biomineralization.^[^ ^25^ ^]^ The optimal duration of OD is however crucial for the generation of in vitro mineralized tissues, where prolonged preculture durations lead to more mature differentiation and higher mineral deposition.^[^ ^22^ ^]^ However, the timeframe required to attain satisfactory levels of mineralization and differentiation varies depending on the cells and the biomimetic culture employed. This leads to culture times often ranging from several weeks to months, which makes it difficult to discern the most critical parameters in obtaining and controlling humanized bone tissue formation, and how important they respectively are.^[^ ^8^, ^22^ ^]^

Currently, there is limited understanding of the impact of preculture conditions on in vivo ectopic bone formation. Evidence collected by previous studies using different biomaterials have demonstrated that in vitro cell differentiation prior to implantation yield enhanced in vivo ectopic bone formation due to the deposition of bone ECM components. This includes collagen, and proteins involved in mineralization, such as alkaline phosphatase (ALP).^[^ ^8^, ^21^, ^26^ ^]^ These findings indicate that cellular ECM constructs are an effective approach for creating implants that offer adjustable physical properties along with bone tissue‐specific biological signals. Based on this evidence, we hypothesized that the degrees of in vitro cellular differentiation and bone ECM deposition prior to implantation, as obtained by optimized preculture conditions, provide strong control over human ectopic BTE outcomes. Conversely, studies have shown that advanced differentiation stages can result in substantial ECM deposition, which may subsequently diminish osteoblastic activity.^[^ ^27^ ^]^ Hence, it is critical to ascertain the precise differentiation and mineralization levels required for in vitro constructs prior to in vivo implantation.

This study elucidated the effect of cellular differentiation and mineralization levels of in vitro microtissues on in vivo bone formation, composition, and structure, by adjusting two highly relevant biomimetic culture approaches; variation of the biomimetic culture composition and variation of its duration. The hypothesis was investigated by two distinct but established BTE materials; CaP‐mPCL microporous scaffolds to represent a mineral ECM‐mimicry strategy and GelMA hydrogels, representing a protein ECM‐mimicry strategy. The quality of the resulting in vivo bioengineered bones from both advanced materials in a standard ectopic mice model was assessed and discussed based on several established parameters, i.e., bone volume, mechanical properties, cellular and extracellular composition, and microstructure, revealing key insights into the choice of biomimetic cultures for control of BTE‐driven human bone formation.^[^ ^9^, ^28^, ^29^ ^]^

## 2 Results

### 2.1 Mineralization Medium Leads to Higher In Vitro Mineralization and Osteogenic Differentiation Compared to Osteogenic Medium

The humanization of rodent models is a sensible way to address the limitations of using animals to study human disease^[^ ^9^, ^13^, ^30^, ^31^, ^32^, ^33^, ^34^, ^35^, ^36^ ^]^ and great progress in in vivo humanized bone models specifically has been achieved in recent years.^[^ ^9^, ^13^, ^37^ ^]^ Yet, it is still currently unclear which, and how, approaches lead to different levels of successful bone tissue formation.^[^ ^38^ ^]^ In this study, we determined which parameters, namely biomimetic culture composition and preculture duration options, were crucial for in vivo humanized BTE and sought to decipher underlying principles. To robustly explore those parameters, two different biomaterials with established osteogenic capacity were used; CaP‐mPCL scaffolds and GelMA hydrogels.^[^ ^9^, ^16^, ^22^ ^]^

**Figure 1:**
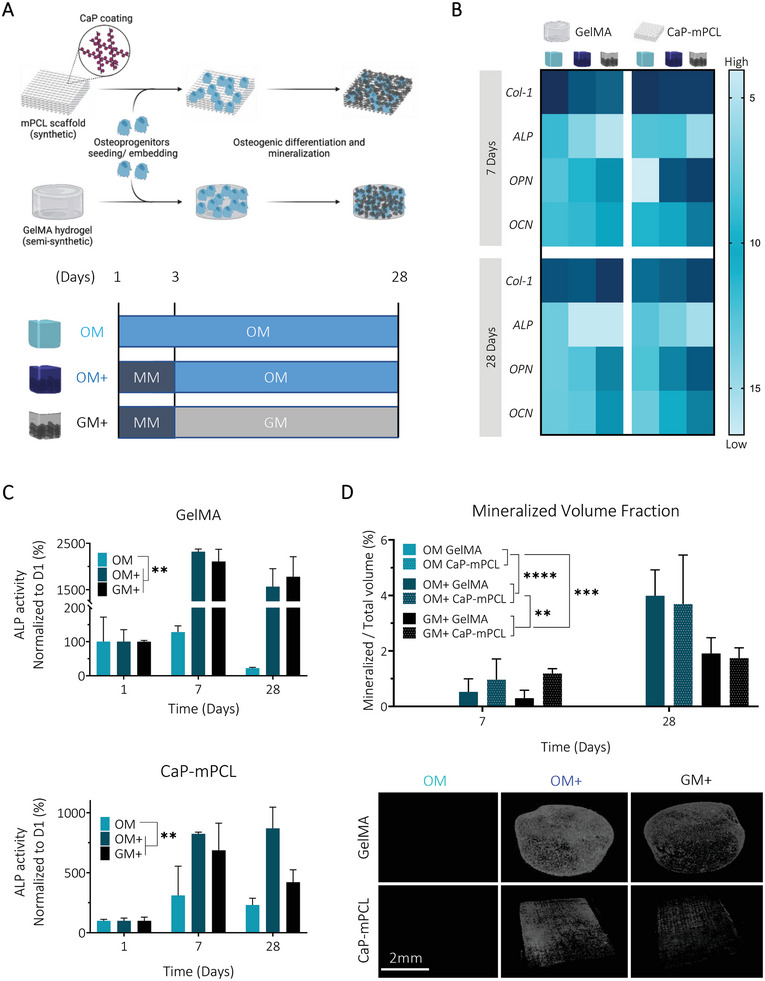
Osteogenic culture medium combined with an initial mineralization medium treatment leads to higher mineralization and osteogenesis in both biomaterial groups (CaP‐mPCL and GelMA). A) Schematic of the experimental design. Primary human osteoprogenitors were seeded on CaP‐mPCL scaffolds or embedded in GelMA 5% w/v hydrogels and were cultured for up to 28 days in OM or in mineralization medium for three days followed by osteogenic medium (OM+) or growth medium (GM+). B) Mean *ΔCq* heatmap from RTqPCR analysis, normalized to the geomean of *7SL* and *RPL32* housekeeping genes. Light blue represents lower mRNA expression (i.e., higher *ΔCq*) and dark blue represents higher mRNA expression (i.e. lower *ΔCq*). C) Alkaline phosphatase activity, normalized to DNA content and to day one. D) Representative images of microcomputed tomography after four weeks of culture and quantification of the mineralized volume fraction. Graphs: Mean + SD, *n* = 3, General Linear Model (Univariate), **p < 0.01, ***p < 0.001, ****p < 0.0001.

First, since the level of cell differentiation in in vitro culture has been proposed as an important criterion for the success of in vivo tissue‐engineered models,^[^ ^8^ ^]^ constructs loaded with primary human osteoprogenitors were cultured in vitro for either one or four weeks in OM, OM with a three‐day mineralization treatment (OM+), or basal growth medium with the same three‐day mineralization treatment (GM+, **Figure** **1**A). We hypothesized that longer‐term ‘preculture’ (referring here to in vitro culture prior to in vivo implantation) and enhanced mineralization would further lead to superior bone formation in subsequent in vivo studies due to potentially superior differentiation capacity.^[^ ^8^, ^25^ ^]^ We also tested the hypothesis that combining traditional OM with a three‐day mineralization treatment (OM+ condition) would lead to higher OD and mineralization compared to OM alone, especially after four weeks of in vitro culture. It was previously shown that this method provided useful matrix cues to bone cells loaded in collagen‐based models and enhanced OD.^[^ ^25^ ^]^

We found that the three‐day mineralization treatment did not impact significantly metabolic activity compared to traditional OM for both materials (Figure S1A, Supporting Information). Prior to implantation, CaP‐mPCL scaffolds showed significantly higher DNA content retained compared to GelMA due to expected increased in vitro proliferation rates on hard fibers compared to soft hydrogels (Figure S1B, Supporting Information). Yet, this DNA content difference did not lead to mineralization differences between both materials, potentially revealing reduced OD degree between both materials. Additionally, DNA content was similar between OM and OM+ in both materials for both preculture durations, which confirmed that OM+ was not impacting cell viability over time. Although metabolic activity increased during the first 2–3 weeks in all conditions and both biomaterials, no significant increase was observed at DNA level over time. These observations indicated that metabolic changes were due to cell differentiation and not proliferation, as could be expected.^[^ ^39^ ^]^ This was confirmed by assessing in vitro OD at the gene level using established early (collagen type‐1 (*Col‐1)*, *ALP*) and late (ostepontin (*OPN)*, osteocalcin (*OCN*)) osteogenic markers.^[^ ^28^, ^40^ ^]^ Despite strong biomaterials differences, namely the use of protein‐driven ECM‐mimicry (GelMA) against a mineral‐driven ECM‐mimicry (CaP‐mPCL), a remarkably similar pattern in gene expression between GelMA and CaP‐mPCL microtissues was observed (Figure 1B). In particular, a similar decreasing trend in expression levels of the early markers *ALP* and *Col‐1* was observed in all conditions, especially in OM+, as would be expected for successful OD.^[^ ^41^ ^]^ However, at the protein level, significantly higher ALP activity was observed overtime in OM+ and GM+ (the basal growth medium without osteogenic supplements, but which received the three‐day mineralization treatment, Figure 1C), with a higher ALP activity from GelMA hydrogels compared to CaP‐mPCL scaffolds (*p* < 0.05). These results are in line with OD where elevated ALP activity is reported in mature osteoblasts and is positively correlated with mineralization rate.^[^ ^41^, ^42^ ^]^ For late osteogenic markers (*OPN*, *OCN*), higher expression was observed for OM+ and GM+ after only seven days of culture, and were stable overtime compared to the OM control (Figure 1B). These results confirmed higher osteogenesis when using an initial and temporary mineralization medium, compared to the OM control traditionally used in the field, with higher levels of late bone markers observed after 7 and 28 days of culture, in line with OD.^[^ ^40^, ^41^ ^]^ The uptake of calcium, one of the main components used for mineralization, was higher overtime in OM+ (*p* < 0.0001) for both materials (Figure S1C, Supporting Information). A significant difference in calcium uptake was identified between both materials (*p* < 0.0001), with lower calcium uptake from CaP‐mPCL scaffolds compared to GelMA hydrogels, as for ALP activity, which may imply less OD, and/or less mineral deposition from mature osteoblasts.^[^ ^43^, ^44^ ^]^ Calcium uptake positively correlated with mineralization levels, as higher mineral volume fractions were observed in OM+ and GM+ compared to control (OM). In particular, higher mineral fraction in OM+ were observed after four weeks of culture compared to GM+, confirming the superiority of using osteogenic supplements (Figure 1D). The results are in agreement with our previous study demonstrating the preliminary efficacy of combining mineralization medium treatment with osteogenic medium (OM+ condition) on GelMA hydrogels.^[^ ^16^ ^]^ We also confirmed that the mineralization treatment induced significantly higher mineralization compared to OM in cell‐loaded gels, even after 12 weeks of culture in vitro (Figure S2, Supporting Information). These results clearly showed that supplementing osteogenic culture media with osteopontin and CaP (some of the components of the mineralization medium) at the early stage of culture is favorable to enhance in vitro biomineralization as shown in other types of materials.^[^ ^25^ ^]^ Despite being reported as a mineralization inhibitor,^[^ ^45^ ^]^ the addition of osteopontin in the mineralization medium serves as a nucleation inhibitor, preventing calcium and phosphate precipitation in the culture medium, and as an intrafibrillar mineralization directing agent,^[^ ^25^ ^]^ leading to better mineral deposition.

Taken together, these findings confirm the hypothesis that longer‐term (four weeks instead of one week) in vitro preculture in OM, combined with three days of mineralization medium supplementation, led to a higher degree of both OD and mineralization, without significantly affecting cell viability. Culturing cells for only one week in OM+ led to higher mineral deposition compared to culturing cells for four weeks in OM, suggesting the superiority of the ‘biomimetic culture composition’ parameter against the ‘preculture duration’ parameter. Strikingly, these results were mostly independent of the ECM‐mimicking approach for the chosen biomaterial (GelMA versus CaP‐mPCL), suggesting a stronger influence of biomimetic culture composition and preculture duration over biomaterial type, as important experimental design parameters.

### 2.2 In Vitro Biomimetic Culture Composition and Preculture Duration Significantly Influence Bone Organ Formation In Vivo

While osteoprogenitors are commonly used for ectopic BTE, a variety of preculture approaches are currently trialed, with no standardized protocol, where cells can be implanted immediately after isolation, after in vitro expansion or after in vitro OD.^[^ ^1^ ^]^ Based on established findings, showing a positive correlation between in vitro osteogenic predifferentiation and bone formation in vivo,^[^ ^8^, ^17^ ^]^ we hypothesized that the level of OD and mineralization of the in vitro construct, at the time of implantation, could be the most determining factor for humanized bone tissue formation and development into a bone organ. Therefore, we tested two different approaches toward controlled and superior mimicry of bioengineered bone ECM; different in vitro biomimetic culture compositions, and different in vitro preculture durations. We hypothesized that a more mineralized osteoblastic microtissue at the time of implantation would lead to a greater in vivo bone formation with higher trabecular network, even in two dissimilar biomaterials, due to the enhanced degree of cell differentiation and mineralization, as well as intrinsic matrix cues harbored by the biomaterials themselves.

**Figure 2:**
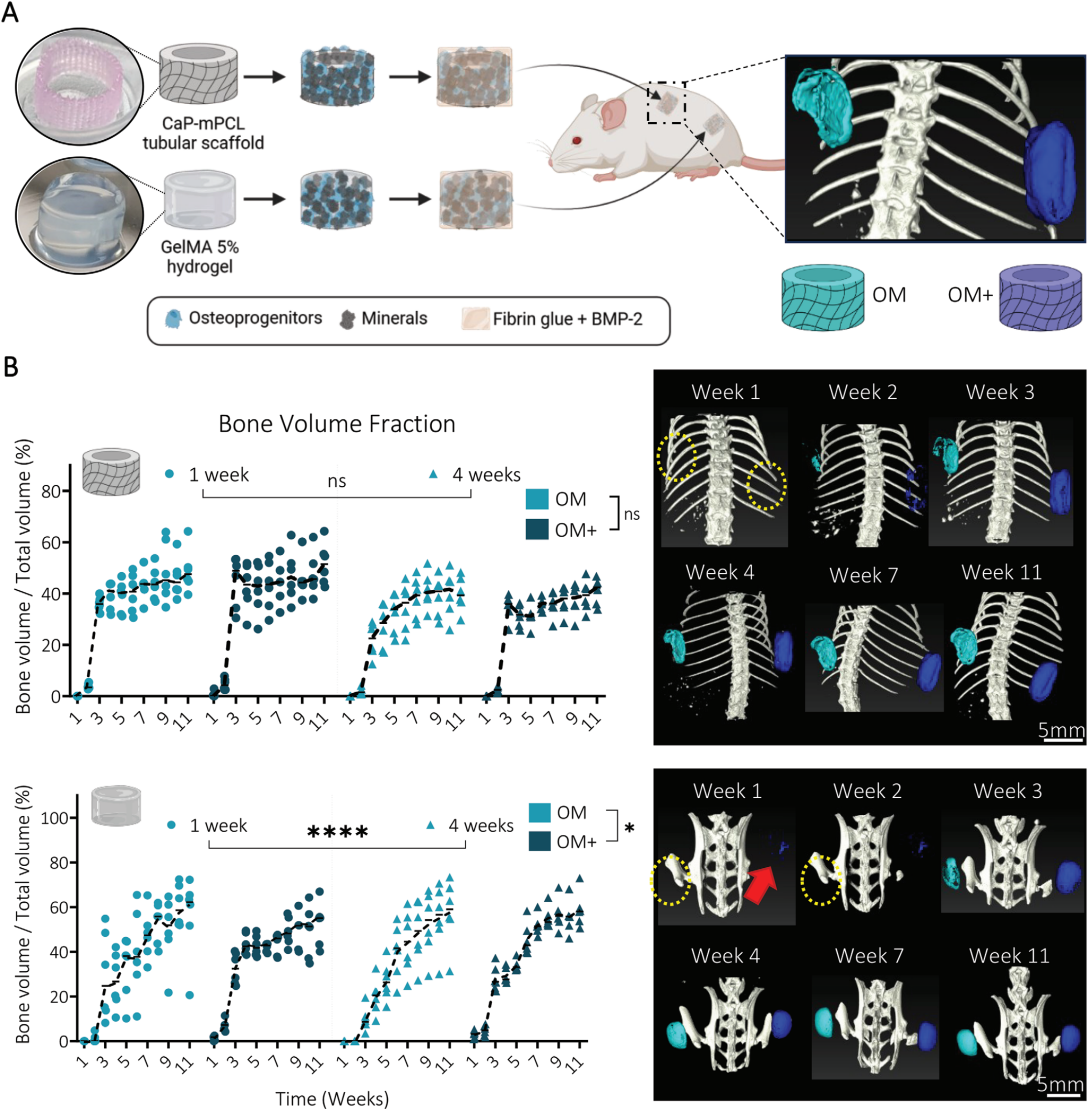
In vivo mineralization of the ectopically formed bone organ from semi‐synthetic GelMA hydrogels was significantly affected by biomimetic culture composition and preculture duration. A) Experimental design: CaP‐mPCL scaffolds and GelMA 5% hydrogels loaded with primary human osteoprogenitors and cultured for four weeks in OM or OM+ prior subcutaneous implantation into immunodeficient mice. B) Quantification and representative images of in vivo mineralization from µCT analyses. Yellow circles represent the location of undetectable implants, red arrow indicates the GelMA OM+ mineralized implants. Median plots with data points and trend line, n = 6, General Linear Model (Univariate), ns: not significant, *p < 0.05, ****p < 0.001.

Based on the in vitro results, both CaP‐mPCL‐derived and GelMA‐derived constructs were precultured in conditions resulting in different combinations of OD and mineralization degrees: 1) low OD / low mineralization (OM for one week), 2) mild OD / low mineralization (OM for four weeks), 3) high OD / mild mineralization (OM+ for one week) and 4) high OD / high mineralization (OM+ for four weeks). Following in vitro culture, the constructs were embedded in fibrin glue loaded with bone morphogenetic protein 2 (BMP2) and subcutaneously implanted in immunocompromised mice flanks (**Figure** **2**A) as previously established.^[^ ^9^, ^16^ ^]^

One‐week post‐implantation, compared to CaP‐mPCL constructs, only the GelMA‐derived constructs previously cultured in OM+, regardless of preculture duration, were detectable (red arrow in Figure 2B; Figures S3 and S4, Supporting Information). Critically, despite both construct types showing similar OD and mineralization levels following in vitro culture and prior to implantation (Figure 1), in vivo mineralization levels were significantly higher for GelMA‐derived constructs as early as one week post implantation (Figure 2B; Figures S3 and S4, Supporting Information). Importantly, we also implanted a small cohort of constructs without BMP2. They failed to form mineralized bone tissue in vivo (Figure S5, Supporting Information), suggesting that mineralized microtissues without exogenous growth factor supplementation were not sufficient to develop and maintain an ectopic bone organ, as could be inferred by our past validation of BMPs use in this context.^[^ ^9^ ^]^ Similar observations were made using mineralized collagen hydrogels, where only mineral deposition was detected, but no proper ossification was created after four weeks in vivo.^[^ ^25^ ^]^ However, here, GelMA‐derived ossicles were the only group showing in vivo mineralization without the need for BMP2 supplementation during the first three weeks following implantation, yet which resorbed after five weeks (Figure S5A, Supporting Information). This may be explained by the sequestration of osteogenic growth factors (GF) produced by the pre‐differentiated cells within the hydrogel, stimulating mineralized bone formation in vivo.^[^ ^46^ ^]^ Yet, those sequestered GF amounts might not be enough to maintain in vivo mineralization over time.^[^ ^47^ ^]^

From samples implanted with BMP2, which compose the core of the study, OM+ derived ossicles showed smoother mineralized bone formation compared to OM derived microtissues, especially in CaP‐mPCL scaffolds (Figures S3 and S4, Supporting Information). Bone volume fraction showed a drastic increase between the second and third week following implantation, especially in OM+, and then stabilized for the CaP‐mPCL‐derived implants, while the GelMA‐derived ossicles continued to form mineralized bone up to week nine (Figure 2B). Overall, significant differences in bone formation over time between biomimetic culture types (*p* < 0.05) and preculture durations (*p* < 0.0001) were only observed in GelMA hydrogels, with slower in vivo mineralization in OM compared to OM+, which could be due to the lower osteogenic predifferentiation of the implanted cells (Figure 1) as seen previously.^[^ ^1^, ^8^, ^26^ ^]^ Slower mineralization rate was observed in GelMA hydrogels compared to CaP‐mPCL scaffolds, which could be explained by the additional hydrogel degradation step during in vivo bone formation,^[^ ^48^ ^]^ or by a possible hypoxic environment within the GelMA hydrogel, leading to chondrogenic mineralized bone formation.^[^ ^49^ ^]^

**Figure 3:**
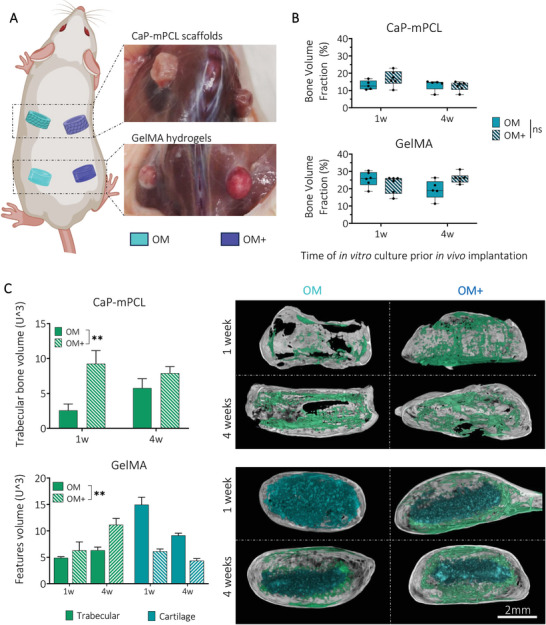
OM+ biomimetic culture leads to similar mineralized bone volume fraction, yet higher trabecular network formation in vivo compared to OM culture. A) After 11 weeks in vivo, mineralized tissues were collected and B) analyzed using µCT. Boxplots, min to max with all data points, n = 6, General Linear Model (Univariate), ns = not significant. C) Quantification and representative images of trabecular and cartilage ossifications from µCT reconstructed images. Bar plots, mean ± SE, n = 6, **p < 0.01.

After 11 weeks in vivo, the bioengineered bone ossicles were excised and collected (**Figure** **3**A), and mineralization degree and tissue morphology were measured using ex vivo microcomputed tomography (µCT). As expected, because samples implanted without BMP2 failed to generate mineralized bone tissue in vivo (Figure S5, Supporting Information), only bioengineered bone tissues derived from constructs implanted with the addition of BMP2 were further used when investigating our hypotheses. Remarkably, the quantification of the bone volume fraction (mineralized volume/total volume) showed no significant difference overall between ossicles, based on their in vitro preculture durations (one versus four weeks) and culture conditions (OM versus OM+) (Figure 3B). However, overall, GelMA hydrogels showed higher bone volume fraction compared to CaP‐mPCL scaffolds (Figure 3B), as detected in vivo at week 11 (Figure 2B). Higher trabecular network was observed for ossicles cultured in OM+ compared to OM, for both materials (*p* < 0.05 for CaP‐mPCL scaffolds, *p* < 0.001 for GelMA hydrogels) and preculture duration (Figure 3C). In addition, constructs precultured in OM+ for only one week (high OD / mild mineralization) showed higher trabecular network and reduced cartilage tissue compared to constructs precultured in OM for four weeks (mild OD/low mineralization), demonstrating the superior effect of preculture medium composition on preculture duration (Figure 3C). These results are in line with the literature where higher osteogenesis and mineralization prior to implantation correlated with higher trabecular network formation.^[^ ^50^ ^]^ In addition, the presence of cartilaginous‐like tissue was observed in GelMA hydrogel‐derived implants (Figure 3C), which was confirmed by collagen type‐2 staining (Figure S6, Supporting Information).The presence of cartilaginous‐like tissue within GelMA confirms the slower and ‘activated’ stage of the mineralization process in these constructs even after 11 weeks of in vivo culture as compared to CaP‐mPCL scaffolds. This finding in GelMA likely correlates with enhanced remodeling at the center of the bone organ due to the balance between hydrogel degradation and new endochondral bone deposition.^[^ ^51^ ^]^

The data indicates that in vitro biomimetic culture composition significantly affected in vivo mineralization and ultimately bone formation, with higher trabecular network from ossicles derived from pre‐mineralized microtissues (OM+ condition). This was true especially after four weeks of preculture in GelMA hydrogels, compared to unmineralized microtissues (OM condition). In addition, some differences were observed between materials where GelMA hydrogels led to slower but higher mineralization, with mineralization occurring from the outer surface of the construct, compared to CaP‐mPCL scaffolds.

### 2.3 In Vitro Preculture Methods and Biomaterial Types Do Not Significantly Affect the Final Mechanical Properties of the Ex Vivo Bone Organ

After establishing that preculture methods could impact bone formation and network architecture in vivo, we evaluated if the different preculture approaches and materials could affect the mechanical properties of the resulting bioengineered bone organs.

**Figure 4:**
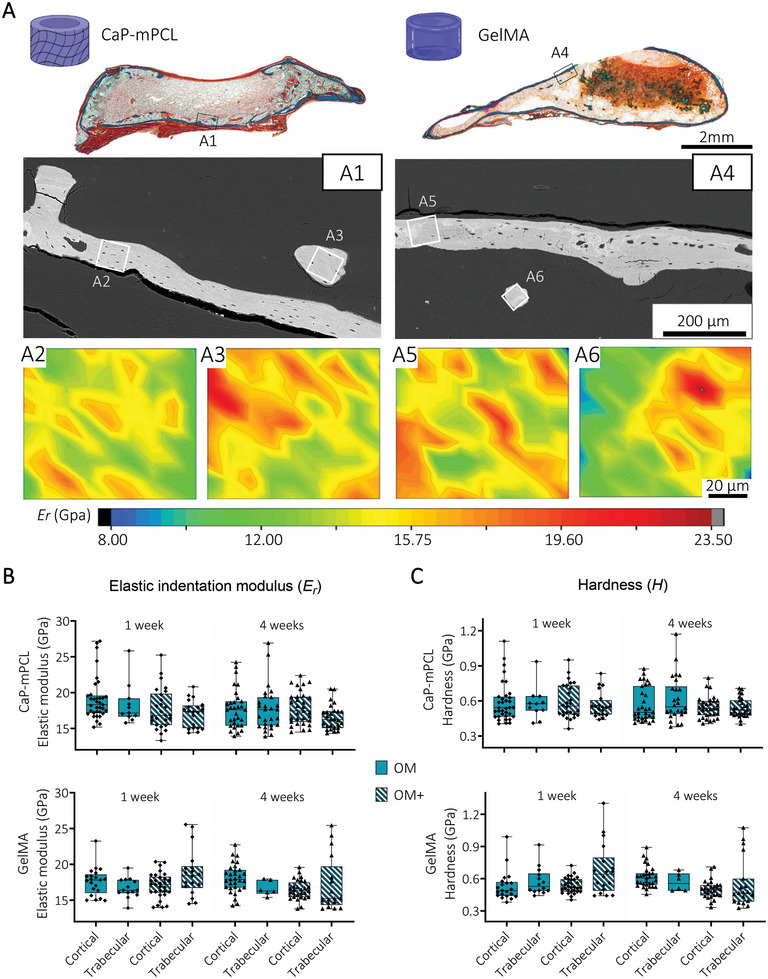
Local mechanical properties of cortical and trabecular bone are similar between bioengineered mineralized tissues. A) Goldner's trichrome staining and BSE images of GelMA and CaP‐mPCL bioengineered mineralized tissues (OM+, 4 weeks preculture represented here) were used to select region of interest (ROI) from cortical (A2, A5) and trabecular (A3, A6) bone for local indentation elastic modulus (*E_r_*) mapping (2D contour maps, color scale representing *E_r_* values). B) Quantification of *E_r_* and C hardness (*H*) from cortical and trabecular regions of the bioengineered bones using depth‐sensing nanoindentation. Box plots (min to max with data points), *n* = 3, average of 15 regions of interest per sample, General Linear Model (Univariate). No significance found (*p*>0.05).

Using Goldner's trichrome staining and backscattered electron microscopy (BSE), specific regions of interest from mature cortical and trabecular ossification throughout the mineralized explants were selected to determine their indentation elastic modulus (*E_r_*) and hardness (*H*) (**Figure** **4**A). Strikingly, no significant differences in elastic modulus were observed between preculture durations, preculture compositions and materials, with an elastic modulus of 17.6 ± 2.4 GPa (mean ± SD) for cortical bone, and 17.0 ± 2.7 GPa (mean ± SD) for trabecular bone (Figure 4B). Similarly, no significant differences in hardness between any of the groups were observed; 0.55 ± 0.12 GPa (mean ± SD) for cortical bone, and 0.57 ± 0.19 GPa (mean ± SD) trabecular bone (Figure 4C). These results are similar to native human femur.^[^ ^52^ ^]^

These findings show that despite using different materials and different preculture methods prior implantation (OM vs OM+, one versus four weeks), the resulting mineralized tissue presented similar mechanical properties, matching native human bone.

### 2.4 Preculture Duration before In Vivo Implantation Influences the Cellular Composition of the Humanized Bone Organ

We next studied the effect of preculture approaches and materials at the cellular level. Here, we hypothesized that preculture duration and composition influenced bone cellular content, leading to a more mature cellular profile, i.e., more toward remodeling instead of a formation phase, in constructs presenting a higher OD and mineralization degree prior to implantation (precultured in OM+ for four weeks).

**Figure 5:**
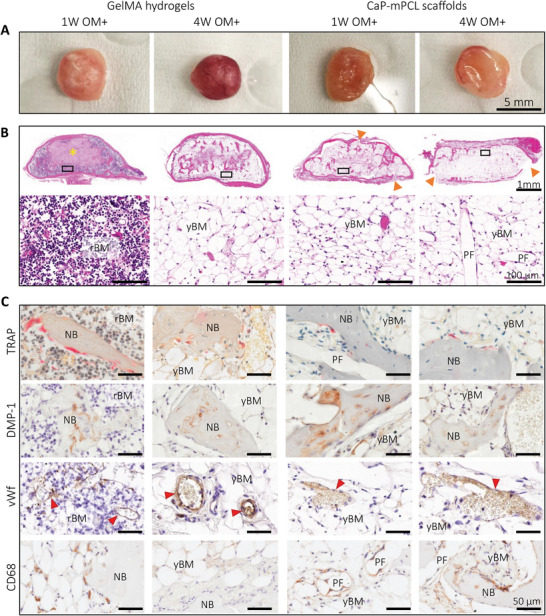
Characterization of the bone cellular matrix from ex vivo samples precultured in OM+ biomimetic culture. A) Macroscopic images of collected explants after 11 weeks of culture in vivo. B) Histology analyses using H&E staining to characterize tissues morphology, ossification and marrow content, with overview (top) and high magnification (bottom). Yellow star: GelMA hydrogel, orange arrows: depleted cortical shell. C) IHC analyses were used to detect osteoclasts (tartrate‐resistant acid phosphatase (TRAP)), osteocytes and chondrocytes (dentin matrix acidic phosphoprotein 1 (DMP‐1)), vascularization (von Willebrand factor (vWf), red arrows) and macrophages (cluster of differentiation (CD68)) using their respective markers. NB: new bone, rBM: red bone marrow, yBM: yellow bone marrow, FT: fibrous tissue, PF: CaP‐mPCL fibers.

Upon tissue collection, 11 weeks post‐implantation (with BMP2), distinct differences in tissue vascularization were already observable, with higher vascularization in four week‐GelMA conditions (in OM and OM+) (**Figure** **5**A; Figure S7A, Supporting Information), compared to GelMA 1 week and CaP‐mPCL explants, for both preculture compositions. Histological analyses (Hematoxylin and Eosin (H&E) staining) confirmed the presence of a cortical shell surrounding every bioengineered bone organ with trabecular structures (Figure 5B). While the cortical shell was very smooth and complete around the bone organs in GelMA‐derived bones cultured in OM+ and in OM‐4 weeks (Figure 5B and Figure S7B), disrupted cortical shell was observed in CaP‐mPCL‐derived bones (Figure 5B and Figure S7B, orange arrows) and GelMA cultured only for 1 week in OM (Figure S7B, orange arrows).

GelMA hydrogels showed significant differences in cell content between preculture duration conditions for both preculture compositions. Remnants of GelMA hydrogel were observed at the center of the tissue (Figure 5B; Figure S7B, Supporting Information; yellow star), confirming the mineralization formation from the outer surface to the center of the construct, as suspected from ex vivo µCT scans (Figure 3). In addition, a difference in bone marrow was evident; red bone marrow (mainly constituted of hematopoietic cells and low percentage of adipocytes (20–40%)^[^ ^53^ ^]^) was detected in tissues that were precultured for only one week (Figure 5B; Figure S7B, Supporting Information), which is characteristic of an hematopoietic marrow found in active bone formation stage, while yellow bone marrow was observed in the four‐week‐GelMA condition (mainly constituted of adipocytes (95%),^[^ ^53^ ^]^ akin to fat marrow, i.e., bone‐maintaining stage)^[^ ^54^ ^]^ (Figure 5B; Figure S7B, Supporting Information). Strikingly, in CaP‐mPCL‐derived explants, no significant difference in marrow content was observed, with yellow marrow in all conditions (Figure 5B; Figure S7B, Supporting Information). However, the resulting bone tissues contained high levels of fibrous tissue within their center, especially when cultured only one week prior to implantation, which indicates intramembranous ossification in CaP‐mPCL‐derived constructs. Bone cells implicated in bone remodeling were detectable within all explants. Osteoclasts (bone‐resorbing cells) were mainly found in regions of resorbing cartilage and around trabecular bone spicules (Figure 5C; Figure S7C, Supporting Information; tartrate‐resistant acid phosphatase (TRAP), pink staining). Mature osteocytes and chondrocytes were found in mineralized bone particles (osteocytes) and mineralized cartilage (chondrocytes) ((Figure 5C; Figure S7C, Supporting Information; with dentin matrix acidic phosphoprotein 1 (DMP‐1), late osteoblastic, osteocyte and chondrocyte marker). Across all conditions, mineralized tissues were all well vascularized, as shown by the positive staining of von Willebrand factor (Figure 5C; Figure S7C, Supporting Information; vWf). Macrophage presence was identified using CD68 marker (M1 and M2 macrophage subset) which was constantly found around the interconnected CaP‐mPCL fibers and within the bone marrow close to bone spicules in all bioengineered bones (Figure 5C; Figure S7C, Supporting Information; CD68). However, lower staining was found in GelMA‐derived bones that were precultured for four weeks, which is in line with mature bone phenotype.^[^ ^28^ ^]^

Throughout the inner cancellous bone, positive cells for human marker *LaminAC* were found in all conditions (Figure S8A, Supporting Information), without any statistical difference except for CaP‐mPCL explants precultured for four weeks in OM as they showed a high proportion of human‐positive fibrous tissue throughout the sample, indicating intramembranous ossification. Indeed, while human cells were mostly found in cartilage and mineralized spicules in GelMA explants, they were mainly within the fibrous tissues found in CaP‐mPCL scaffolds. Conversely, in all bioengineered humanized bones, derived from GelMA or CaP‐mPCL scaffolds, no positive cells for human markers were found in the cortical shell, which could indicate that the cortical bone of the bioengineered ossicle, was derived from the host cells. To confirm this hypothesis, and to ensure that these human cells were not only maintained but also participated to the mineralization process in vivo, murine and human‐specific type 1 collagen antibodies (mCol‐1 and hCol‐1 respectively) were used (Figure S8B, Supporting Information). As observed previously in GelMA‐derived bone explants,^[^ ^16^ ^]^ the cortical shell was only positive for mCol‐1, in both materials. However, within the cancellous bone, ossified tissues were mainly positive for hCol‐1 with some bone spicules showing hybrid between human and murine origin, where mCol1 positive areas were found in cancellous/woven bone particles while hCol1 positive areas corresponded to mineralized cartilage. Despite using an ectopic model to avoid any interference from the host bone cells, subcutaneous implantation stimulates a wound healing response, inducing progenitors’ mobilization in the implantation site, which can thus participate in bone formation.^[^ ^55^ ^]^

Overall, the histological characterization of the bioengineered bones presented here strongly supports that the cellular composition was mainly affected by preculture durations rather than materials or preculture compositions, where longer preculture duration led to a more mature phenotype in vivo (i.e, remodeling rather than mineralization stage). Tissue morphology and cell content were similar between materials, except for the presence of fibroblastic tissue which was only seen in CaP‐mPCL explants. The only distinctive differences observed between culture compositions were seen in GelMA explants where OM+ led to higher vascularization after 11 weeks in vivo compared to OM samples. Thus, in line with our hypothesis, the combination of longer preculture duration (four weeks) and OM+ biomimetic preculture led to a more mature bioengineered bone.

### 2.5 In Vitro Preculture Duration and Biomimetic Culture Composition Affect the Ossification and ECM Deposition Processes In Vivo

The composition of the ECM plays an important role in bone function and structure.^[^ ^56^ ^]^ Because the ECM formation and composition are correlated with cellular composition,^[^ ^57^ ^]^ we hypothesized that preculture duration would be the most determinant factor to obtaining mature ossification and protein deposition as it was for the cellular composition.

**Figure 6:**
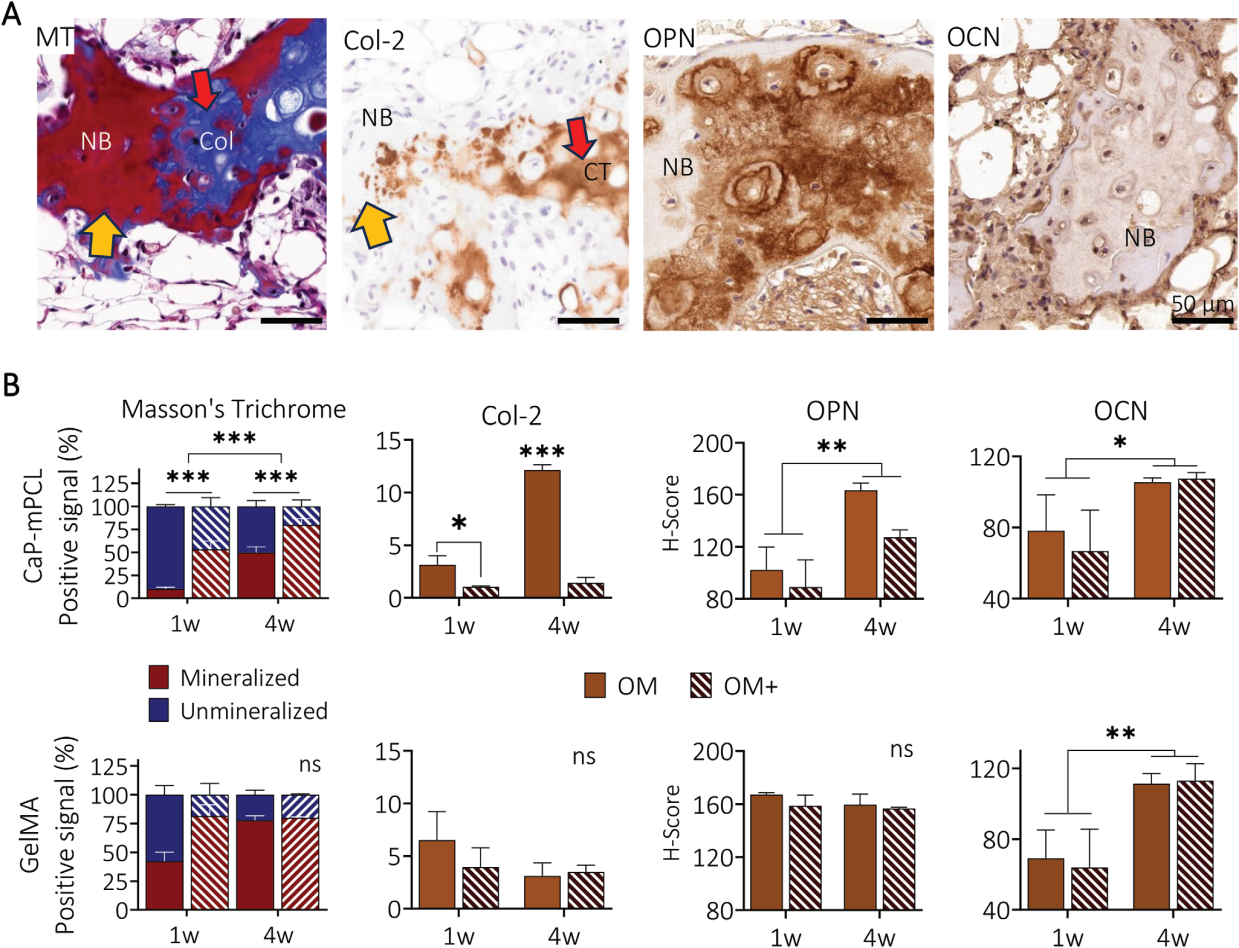
Characterization of bone ossification and extracellular matrix deposition from ex vivo samples. A) Representative images of MT, collagen type 2 (Col‐2) (yellow arrows: mature bone, red arrows: unmineralized collagen deposition, sample: GelMA‐derived sample cultured in OM+ for one week), and the key bone proteins osteopontin (OPN) and osteocalcin (OCN) from GelMA‐derived samples cultured in OM+ for four weeks prior to in vivo implantation. B) Quantification of MT, collagen type 2, osteopontin and osteocalcin from ex vivo samples. Bar plots with mean ± SE, n = 3 biological replicates per staining. General Linear Model (Univariate), ns: not significant, *p < 0.05, **p < 0.01 ***p < 0.001. NB: new bone, Col: unmineralized collagen, CT: cartilage tissue.

Using standard bone assays (i.e, Goldner's and Masson's trichrome (MT), Safranin O/Fast green, collagen type 2 (Col‐2) stains), different stages of ossification were detected in the bioengineered bone ossicles (Figures S9 and S10, Supporting Information). While constructs precultured in OM+ showed mainly mature and mineralized bone spicules and cortical shell (Figures S9A–C and S10A–C, Supporting Information; yellow arrows) with few cartilage islands (Col‐2 positive areas, Figures S9D and S10D, Supporting Information; red arrows), constructs precultured in OM showed more immature bone tissue and the presence of cartilage tissue (Figures S9A–D and S10A–D, Supporting Information; red arrows). Quantification of MT and Col‐2 (**Figure** **6**) confirmed that OM conditions presented higher areas of unmineralized cartilage compared to OM+ conditions in CaP‐mPCL scaffolds for both preculture durations while no significant differences between conditions were observed in GelMA constructs (*p* = 0.067 for MT, *p* = 0.549 for Col‐2). Collagen type 2 confirmed the presence of endochondral ossification, driven by BMP growth factors,^[^ ^58^ ^]^ within the humanized bones derived from both materials, where cartilage formation was affected by the CaP supplementation during the preculture of CaP‐mPCL scaffolds. In addition to ossification maturation, late bone markers such as osteopontin and osteocalcin were detected throughout the bioengineered bones (Figures S9E,F and S10E,F, Supporting Information). Higher levels of osteopontin and osteocalcin were detected in samples precultured for four weeks compared to one week, in CaP‐mPCL scaffolds and in both preculture compositions (Figure 6), which is in line with maturation processes in bone.^[^ ^28^ ^]^ However, in GelMA hydrogels, only osteocalcin deposition was significantly impacted by preculture durations.

**Figure 7:**
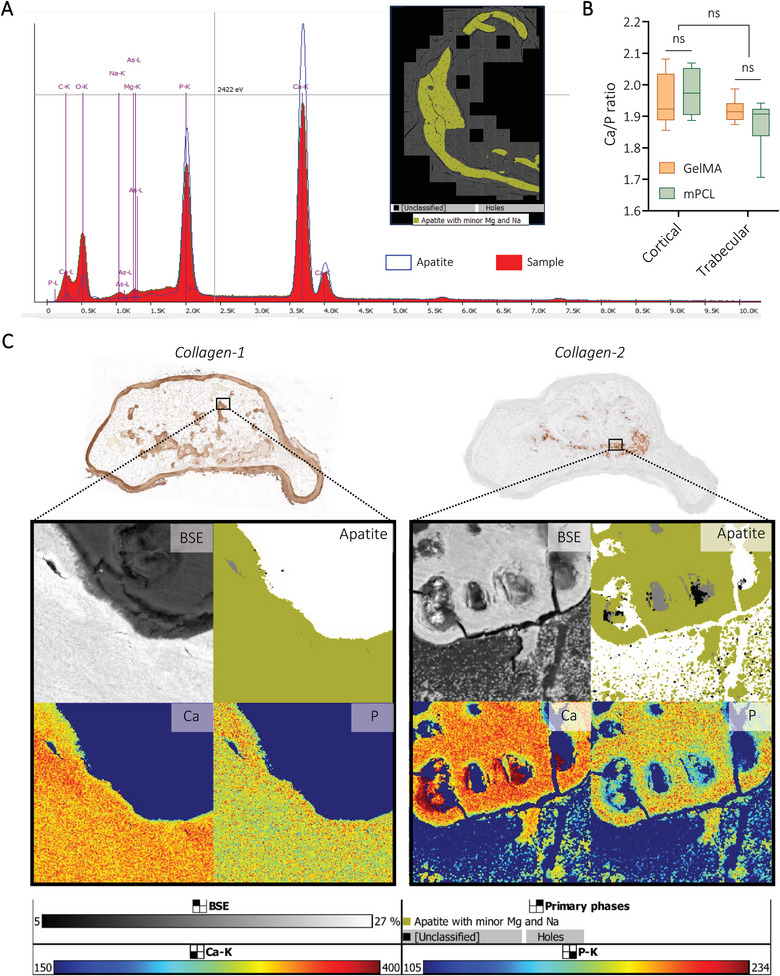
Application of TIMA analyses to determine mineral composition of bioengineered bone tissues. A) Spectra of apatite with minor levels of magnesium and sodium detected using TIMA. B) Quantification of Ca/P ratio using EDS analyses from Tescan MIRA. Box plots (Mean with min to max), *n* = 3, General Linear Model (Univariate), ns = not significant. C) Representative images of explants (GelMA‐derived, precultured for four weeks prior to 11 weeks in vivo) showing the correlation between ECM composition (IHC) and mineralization levels (BSE) with apatite, calcium (Ca), and phosphate (P) distribution.

In addition to standard immunohistochemistry (IHC) techniques, mineralized particles were characterized here using an innovative application of Tescan Integrated Mineral Analyzer (TIMA, usually reserved for geological samples) applied to collagenous mineralized tissues^[^ ^59^ ^]^ to further determine composition. Throughout the humanized tissues, in both GelMA and CaP‐mPCL constructs, mineralized particles were positive for apatite with minor levels of magnesium (Mg) and sodium (Na), as found in the native bone,^[^ ^60^ ^]^ based on the spectral outputs (**Figure** **7**A). Such levels were similar between both construct types. Using the energy dispersive spectroscopy (EDS) function of TIMA, we could detect, map and quantify the deposition of calcium (Ca) and phosphate (P) ions within mineralization. Overall, an average Ca/P ratio of 1.96 ± 0.07 for cortical bone and 1.89 ± 0.06 for trabecular bone were detected in all conditions and for both materials (Figure 7B; Figure S11, Supporting Information). Despite being higher than the reported Ca/P ratio for hydroxyapatite crystals (1.67), measured Ca/P ratios were close to the average Ca/P ratio of native human bones.^[^ ^61^ ^]^ By correlating IHC images for collagen type 1 (Col‐1) and collagen type 2 (Col‐2) with backscattered electron (BSE) imaging and TIMA, correlations between mineralization density and Ca/P ratio were found (Figure 7C). Indeed, in mineralized areas (Col‐1 positive), Ca and P ions were homogeneously distributed and showed an average ratio of 1.92 ± 0.07. On the other hand, mineralizing cartilage areas (Col‐2 positive) showed higher calcium concentrations with lower phosphate deposition, which is relevant to cartilage calcification process during endochondral ossification.^[^ ^62^ ^]^ These areas presented a Ca/P ratio of 2.22 ± 0.05, which is similar to Ca/P ratio found in the femoral bone (average Ca/P ratio of 2.22),^[^ ^61^ ^]^ and in calcified rodent cartilage (average of 2.17), but higher than in human calcified cartilage.^[^ ^63^ ^]^ Apart from the results themselves, our study showcases TIMA as an appropriate method for use in biological samples and offers alternatives to other techniques such as BSE/EDS, X‐Ray diffraction, and atomic forces microscopy techniques, in future analysis of mineralized ECM.

In summary, these results show that in vitro preculture conditions define the ECM composition and maturation of ossification once in vivo, where longer preculture duration and higher level of biomineralization before implantation led to more mature stages of ossification and ECM deposition (higher levels of late bone markers). Maturation stages of ossification directly correlated with Ca/P ratio composing the mineral particles, where mineralizing collagen type 2 depositions showed higher proportion of calcium, as seen in healthy endochondral ossification, which could be controlled by the preculture conditions.

### 2.6 Biomimetic Culture Composition Influences the Organization of Collagen and the Osteocyte Lacunar‐Canalicular Network (LCN)

In addition to its composition, the ECM structure and organization are important parameters for bone quality, where disruptions of collagen fibers organization in adult bone are generally considered as pathologic.^[^ ^57^, ^64^ ^]^ Therefore, analyzing the arrangement of collagen within the mineralized matrix and the organization of the osteocyte lacunar‐canalicular network (LCN) brings complementary information on the quality of the bioengineered humanized bones. Although no significant difference was observed on the overall mechanical properties, in vitro preculture approaches significantly influenced cellular and extracellular composition of the humanized bones. Therefore, we hypothesized that preculture type and duration may impact ECM arrangement as well.

**Figure 8:**
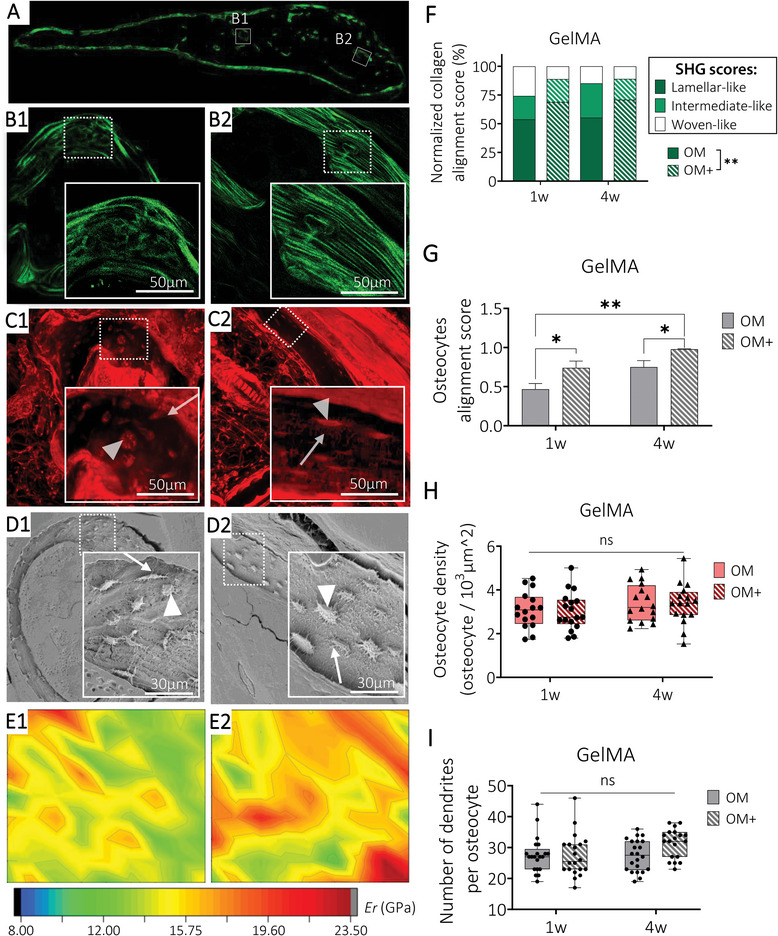
Collagen fibers organization and their relationship with osteocyte LCN and mechanical properties. Representative ROI images from GelMA bioengineered humanized bone with one week of preculture in OM+ condition of A‐B) second harmonic generation imaging collagen fibers, C) laser confocal scanning microscopy imaging LCN from rhodamine‐stained samples and D) Scanning electron microscopy of acid etched samples detecting osteocytes embedded in the mineralized matrix. Osteocyte body indicated by arrowheads (Δ) and canaliculi indicated by arrows (↗). E) Indentation elastic modulus maps of the corresponding ROIs. Semi‐quantification of F) collagen alignment score classified into woven‐like (B1), intermediate and lamellar‐like (B2) arrangement (mean, *n* = 3 sample per group), G) osteocytes alignment score (mean ± SE, *n* = 10 ROIs with a total of 190 cells analyzed per condition), H) osteocyte density and I) number of dendrites per osteocytes (box plots, min‐max, *n* = 15‐20 ROIs per condition). General Linear Model (Univariate), ns = not significant, *p < 0.05, **p < 0.01.

Collagen arrangement was detected using second‐harmonic generation (SHG) imaging (**Figure** **8**A) and defined using scores: woven‐like (randomly oriented fibers, immature collagen organization (Figure 8B1), lamellar‐like (parallel fibers, mature collagen organization, Figure 8B2) and intermediate (mix of parallel and randomly oriented fibers).^[^ ^29^ ^]^ Collagen arrangement was not significantly impacted by preculture durations but was influenced by preculture composition, in both materials. Constructs exhibiting high levels of OD and mineralization prior to implantation (OM+, one and four weeks of preculture) showed a higher proportion of lamellar‐like arrangements, corresponding to a mature organization, while unmineralized implanted microtissues (OM preculture) led to higher proportion of woven‐like arrangements (immature collagen deposition, Figure 8F; Figure S12A, Supporting Information). Higher proportions of woven‐like arrangements were detected in GelMA hydrogels compared to CaP‐mPCL scaffolds, even in OM+ conditions, which confirmed the slower maturation of the mineralization process in GelMA conditions. Osteocyte network organization (Figure 8C,D), was correlated with collagen fibers arrangements, where lamellar‐like arrangements showed better osteocyte alignment, following the collagen fiber directions, with perpendicular canaliculi (Figure 8C1–D1 vs Figure 8C2–D2), in line with previous studies.^[^ ^29^ ^]^ The higher ECM organization in OM+ conditions was consistent with the LCN appearance, where better osteocyte alignment was observed in both preculture duration, with higher alignment score from OM+ with four weeks of preculture prior to implantation (Figure 8G; Figure S12B, Supporting Information). Organized regions (lamellar‐like collagen arrangement and aligned LCN) within all the bioengineered humanized bone tissues showed higher elastic modulus (Figure 8E2) compared to disorganized regions (woven‐like arrangement, Figure 8E1), which is in line with observations in native bones.^[^ ^65^ ^]^ Focusing on the LCN, similar osteocyte density (Figure 8H; Figure S12C, Supporting Information) and dendrites number per osteocytes (Figure 8I; Figure S12D, Supporting Information) were observed between preculture conditions in both biomaterials.

All together, these findings demonstrate that in vitro biomimetic precultures impacted the ultrastructure of the bioengineered bones which ultimately affected not only its cellular arrangement but also its local mechanical properties, and therefore its quality.^[^ ^64^ ^]^ Hence, highly mineralized microtissues led to more organized, and therefore more mature ossified structure formation in vivo compared to unmineralized ones.

## 3 Discussion

Despite the prevalence of BTE in regenerative medicine, the absence of standardized protocols for bone bioengineering persists, mainly due to a high variety of co‐dependent parameters influencing successful BTE outcomes.^[^ ^3^ ^]^ Although human primary osteoprogenitors are preferred for BTE constructs,^[^ ^1^ ^]^ there is considerable variability in material selection, preculture techniques, and the inclusion of minerals, proteins, or growth factors.^[^ ^1^, ^3^, ^8^, ^66^, ^67^ ^]^ Nevertheless, the integration of functionalized biomaterials with in vitro biomimetic approaches yields bioengineered bone organs closely resembling native bone across multiple dimensions, including mineral composition, bone marrow functionality, and collagen fiber organization.^[^ ^16^, ^27^, ^29^, ^68^ ^]^ Despite advancements, fundamental questions regarding the critical parameters for accurately mimicking human bone structure and composition at cellular and extracellular levels remain unanswered, prompting this study to investigate the qualitative and quantitative impacts of key parameters in BTE. Based on established knowledge,^[^ ^8^, ^27^ ^]^ we selected in vitro biomimetic ‘preculture composition’ and in vitro ‘preculture duration’ prior to in vivo implantation as key criteria to focus on, as they directly regulate in vitro OD and mineralization.^[^ ^8^, ^69^ ^]^ In order to best investigate those parameters, we conducted a comprehensive study of their impact on the cellular and extracellular composition, ultrastructure and overall properties of humanized bioengineered bone organs, from dissimilar cell‐loaded biomaterials precultured in vitro, namely fibrous CaP‐mPCL scaffolds and GelMA hydrogels (**Table** **1**).

**Table 1:** Summary table recapitulating the impact of preculture durations, preculture compositions, and biomaterials on bioengineered bone tissue parameters.

Bone criteria	Preculture duration	Preculture composition	Biomaterial type
In vivo bone formation	+	+	+
Type of ossification (cortical / trabecular / cartilage)	++	++	+
Overall mechanical properties	=	=	=
Vascularization	+	=	+
Overall structure/morphology	+	++	+
Bone marrow	+	=	+
Cellular composition	+	++	+
Humanization	=	=	=
Ossification maturation	+	++	=
ECM maturation	++	=	=
Collagen fibers organization	=	++	=
LCN organization	+	++	=
Impact score	**11**	**13**	**6**

= means no significant impact, score = 0, + means significant impact in some conditions, score = 1, ++ means significant impact in all groups, score = 2.

Prior investigations have underscored the importance of cellular differentiation of in vitro constructs preceding implantation, with collagen synthesis as a critical determinant for ectopic bone formation.^[^ ^70^ ^]^ Yet these studies have not delineated the optimal degree of in vitro OD and whether this is a critical parameter in BTE in terms of mineralization, ECM composition and ECM organization in vivo. As corroborated by our own findings, it is established that prolonged in vitro differentiation leads to higher osteogenesis and subsequent mineral deposition.^[^ ^22^, ^69^ ^]^ However, as demonstrated previously, depending on their initial osteogenic capacity, differentiated cells can reach a maximum mineralization capacity and therefore trigger an osteolytic and bone remodeling phase after prolonged in vitro culture,^[^ ^69^ ^]^ in addition to increasing ECM deposition obstructing pores,^[^ ^27^ ^]^ which could negatively affect in vivo bone formation. Moreover, the extent of osteoblastic activity in vitro does not consistently correlate with in vivo bone formation.^[^ ^70^ ^]^ Furthermore, the potential impact of exogenous supplementation of growth factors warrants investigation as it could potentially challenge these established observations. It is known that the use of BMPs in BTE allows faster and higher bone formation and enhanced ECM‐mimicry without altering the structural hierarchy of bone,^[^ ^71^ ^]^ even without the combination of osteogenic cells.^[^ ^72^ ^]^ Hence, our study aimed to ascertain whether high levels of OD and mineralization at the time of in vivo implantation indeed represent pivotal factors in replicating more faithfully human bone physiology, assuming BMP2 bolus supplementation at implantation time.

From this study, the level of OD, and therefore mineralization, prior to implantation was shown to be the most critical parameter in ectopic BTE. This was easily modulated using different biomimetic cultures and preculture durations approaches. To differentiate osteoprogenitors such as MSCs into osteoblastic cells, a common method is the use of OM, where proteins (dexamethasone and ascorbic acid) and inorganic phosphate ions (β‐glycerophosphate) activate *RUNX2* expression (early osteogenic marker, essential for osteoblast differentiation) via activation of the *WNT/βcatenin* signaling pathway, collagen and hydroxyapatite production and the regulation of BMP2 production.^[^ ^73^ ^]^ However, to obtain mature osteoblast differentiation and high mineral deposition from primary human osteoprogenitors, a long culture duration is required, usually between 7 to 13 weeks, depending on the initial osteogenic capacity of the donor cells.^[^ ^69^ ^]^ In this study, the assessment focused on the level of OD and mineralization to generate in vivo ectopic bone formation. Hence, to ensure that the osteogenic cells remained within the linear phase of differentiation and mineralization, we subjected the constructs to preculture duration of either one or four weeks. This approach aimed to prevent the cells from progressing to a more advanced stage of maturation. As demonstrated previously on collagen‐based hydrogels,^[^ ^25^ ^]^ enhanced in vitro OD and mineral deposition were obtained from a calcium and phosphate supersaturated medium (mineralization medium, MM) at an early stage of in vitro 3D culture (first three days of OD and mineralization induction). This was shown for the first time here for CaP‐mPCL‐derived constructs and validated again with GelMA after our preliminary results,^[^ ^16^ ^]^ hence acting regardless of the type of biomaterial. While the mechanisms of OD activation from OM supplements are well‐known,^[^ ^73^ ^]^ the biomolecular mechanisms involved in the CaP‐mediated OD are still not fully understood. Evidence has shown that the combination of calcium and phosphate ions can trigger OD through the same regulation pathway as dexamethasone (BMPs/SMAD‐ and RAS/RAF/ERK‐dependent signaling pathways), in addition to inhibiting some microRNAs expression involved in bone remodeling, resulting in increased OD and enhanced ectopic bone formation.^[^ ^74^ ^]^ In addition to the physical cues provided by the CaP‐enriched microenvironment, mimicking the natural inorganic environment of bone, Shih et al. also demonstrated the effect of phosphate‐enriched extracellular milieu on the activation of adenosine signaling, via ATP synthesis, promoting OD of MSCs.^[^ ^75^ ^]^ The observed enhanced bone tissue formation in vivo from highly mineralized in vitro microtissues was also observed in various studies using functionalization of biomaterials with mineral phases (HA, CaP, β‐TCP).^[^ ^3^, ^8^, ^24^ ^]^ However, supplementing the culture media with a mineralization medium treatment for three days is an advantageous approach compared to biomaterials functionalization as it is a simple and cost‐effective method, which does not require specific knowledge in composite biofabrication and does not alter the viability of the encapsulated cells. Although rapid mineralization has been shown to potentially yield suboptimal bone quality, as observed in bone fracture healing processes,^[^ ^57^ ^]^ here the rapid mineralized bone formation induced by pre‐mineralized microtissues resulted in superior organization of collagen fibers and osteocytes compared to OM alone. This implies that an approach centered on mineral supplementation combined with osteogenic factors is a superior method for more faithfully mimicking the native bone organ composition and structure by inducing OD, and therefore mineral deposition, through multiple pathways. Thus, if the goal is to rapidly create a model with mature mineralization and ultrastructure in vivo, OM+ is the preferred biomimetic approach, even for just one week of in vitro preculture. However, if time is not a critical BTE aspect, a longer preculture duration will combine high mineralization levels with a more mature ECM organization and composition. Hence, OM+ offers a robust solution to generate mineralized bone organs more rapidly, with superior quality compared to the conventional OM approach.

Another crucial finding in this study is the superiority of preculture conditions against the type of biomaterial chosen, despite the use of vastly different biomaterials, where cells are embedded in a soft matrix (GelMA hydrogel) or seeded on hard fibers combined with a mineral ECM‐mimicry approach (CaP‐mPCL scaffold). This can be explained by the similarities shared by both materials. In addition to supporting cell attachment, growth and osteogenesis, both materials presented pore size favorable for osteogenesis, bone formation, and vascularization (pores >300 µm; average of 370 µm for GelMA 5%,^[^ ^76^ ^]^ and 450 µm for CaP‐mPCL).^[^ ^77^ ^]^ These characteristics are considered crucial material parameters for BTE.^[^ ^2^ ^]^ Moreover, both biomaterials presented ECM mimicry approaches, which, despite being from different nature (protein ECM‐mimicry for GelMA, mineral ECM‐mimicry for CaP‐mPCL), support osteogenesis.^[^ ^2^, ^5^ ^]^ Another important similarity between both materials was their levels of mineralization and OD from each preculture approach prior to implantation, which, as explained previously, significantly affect in vivo bone formation.^[^ ^8^, ^17^ ^]^

Importantly, from this study, GelMA emerged as a favored biomaterial for investigations related to bone biology and developmental studies due to its ability to generate bioengineered bone tissues exhibiting diverse bone stages (i.e., bone formation or bone remodeling) within the timeframes chosen for this study (11 weeks in vivo). Conversely, observations regarding CaP‐mPCL scaffolds revealed accelerated bone formation kinetics accompanied by fibroblastic tissue deposition, mimicking the supportive collagen matrix deposition seen in bone fracture healing.^[^ ^78^ ^]^ This justifies its existing use for application in bone regenerative medicine, e.g., for load‐bearing and long bone defects in particular^[^ ^79^ ^]^ which require fast healing. Understanding how and why different bone marrow stages could be obtained from GelMA and not from CaP‐mPCL scaffolds could reveal important pathways in ectopic bone development. One hypothesis explaining these differences could be the slower bone formation obtained from GelMA precultured for only one week in vitro, which still shows an active endochondral bone formation stage and hydrogel degradation, through enzymatic degradation enhanced by its matrix metalloproteinase degradation sequences,^[^ ^15^ ^]^ within its center after 11 weeks in vivo. Therefore, the presence of hematopoietic bone marrow (found in bone formation stage) in constructs precultured for only one week could be explained by the recruitment of hematopoietic and inflammatory cells to promote OD, and therefore initiate bone formation, by altering the Notch signaling pathway and secreting factors such as chemokines (IL‐6, VEGF) and BMPs, as seen in bone development or bone fracture healing.^[^ ^80^ ^]^ Conversely, faster in vivo bone formation observed in CaP‐mPCL scaffolds or from highly mineralized GelMA microtissues, resulted in a mature bone phase after 11 weeks, with fatty bone marrow (found in bone remodeling stage), where bone marrow fat plays an important role in bone homeostasis.^[^ ^81^ ^]^ By further optimizing this aspect, GelMA hydrogels could potentially be used for a broad spectrum of bone research applications.

Whilst current BTE models using 3D constructs loaded with pre‐differentiated osteoprogenitors, such as the one presented in this study, successfully replicate some of the ECM composition of native bone, current limitations include restricted ability to fully recapitulate the full human cellular bone compartment (with only 50–60% of the cellular compartment positive for human markers on average).^[^ ^10^ ^]^ Employing osteoprogenitors pre‐differentiated into osteoblasts leads to the formation of a hybrid human‐murine bone organ with bone marrow compartment derived from host cells. However, recent investigations are exploring the development of humanized bone organs integrating human hematopoietic and vasculature cells to achieve a more comprehensive mimicry of the intricate bone microenvironment.^[^ ^13^, ^32^, ^37^ ^]^ These efforts not only enhance the physiological relevance of bone tissue models but also provide valuable platforms for studying human‐specific bone diseases. Yet, this increase in complexity needs to be moderated by a thorough study of the parameters involved to understand underlying principles and therefore control their tailoring.

## 4 Conclusion

By employing two fundamentally different biomaterial and ECM mimicry approaches, we have demonstrated in an ectopic mouse model the commending capacity of in vitro preculture methods prior to implantation to generate in vivo humanized ectopic bone tissues that closely mimic the human organ. Combining a mineralization treatment with osteogenic medium (OM+) proved to be a highly successful method to enhance OD and biomineralization in vitro, leading to the faster in vivo formation of tissue‐engineered bone organs that exhibit superior mineralization, trabecular network, ossification maturity, structural organization, and extracellular composition compared to the use of OM alone. OM+ not only facilitates the generation of more mature humanized bone organs, but also allows researchers to reduce preculture duration before implantation. Manipulating the level of cellular differentiation and mineralization in vitro prior to implantation yielded varying levels of bone marrow maturation from GelMA hydrogels, demonstrating GelMA's capability to achieve diverse stages of cartilage and bone development within the 11 weeks of study, whilst CaP‐mPCL scaffolds promoted rapid mature bone formation through endochondral and intramembranous ossification processes. Future advancements in ectopic BTE may involve enhancing current models by incorporating human hematopoietic, immune, and vascular progenitors to increase the mimicry level of human bone, thereby varying their utility as diverse preclinical models.

## 5 Experimental Section

### Osteoprogenitor Isolation and Culture

Human primary osteoprogenitors (pOBs, QUT Human Research Ethics Committee, approval number 1400001024) were isolated from bone chips collected during hip replacement or knee surgeries from male donors, as previously described,^[^ ^82^ ^]^ and cultured in basal growth medium (GM, **Table** **2**). To create 3D human osteoblast‐derived microtissues (hOBMs), human pOBs were used at passage 3–4. Routine mycoplasma testing was performed during in vitro culture (cultures were negative for mycoplasma).

**Table 2:** Cell culture media composition.

Media	Components
Basal growth medium (GM)	αMEM (Gibco), 10% (v/v) fetal bovine serum (FBS, ThermoFisher), 1% (v/v) Penicillin/Streptomycin (P/S) (ThermoFisher)
OM	αMEM, 10% (v/v) FBS, 1% (v/v) P/S, Ascorbate‐2‐phosphate (50 µg mL^⁻1^, Sigma‐Aldrich), β‐Glycerophosphate (10 mm, Sigma‐Aldrich), Dexamethasone (100 nm, Sigma‐Aldrich)
Mineralization medium (MM)	αMEM, 10% (v/v) FBS, 1%(v/v) P/S, milk‐extracted osteopontin (100 µg mL^−1^, a gift from Arla Food), calcium chloride dihydrate (9 mM, Sigma‐Aldrich), dipotassium phosphate (4.2 mM, Sigma‐Aldrich), HEPES (25 mM, Sigma‐Aldrich)

### Bioengineering of 3D Human Osteoblast‐Derived Microtissues (hOBM)—Cell Encapsulation in GelMA Hydrogels

Gelatin methacryloyl (GelMA) 5% (w/v) precursor solution was generated using sterile GelMA stock solution (10% (w/v), porcine Type A, 300 bloom, 80% degree of methacrylation, Gelomics Pty Ltd, Brisbane, QLD, Australia) diluted in Phosphate Buffer Saline (PBS) to a final concentration of 5% (v/v) and mixed with the photoinitiator I2959 (1‐[4‐(2‐hydroxyethoxy)‐phenyl]‐2‐hydroxy‐2‐methyl‐1‐propanone, 0.05% (w/v) BASF, Ludwigshafen, Germany). The human pOBs were resuspended in pre‐warmed GelMA 5% (w/v) precursor solution (37°C) at a cell density of 2.2 × 10^6^ cells per mL. As previously described,^[^ ^16^ ^]^ 65 µL of cell‐loaded precursor solution was transferred in each well of a sterile mold (custom‐made polytetrafluoroethylene casting molds, wells of 3 mm depth and 5 mm diameter, QUT Design and Fabrication Research Facility, Brisbane, QLD, Australia), covered with a glass slide and crosslinked for 15 min at 365 nm, with an intensity of 2.6 mW cm⁻^2^ (crosslinker CL‐1000 L, UVP, Upland, CA, USA). Once crosslinked, the cell‐loaded gels were transferred into 48‐well plates using a sterile spatula, washed in PBS for 10 min at 37 °C and cultured in 1 mL of GM in a humidified incubator (37 °C, 5% CO_2_) until OD and mineralization.

### Bioengineering of 3D Human Osteoblast‐Derived Microtissues (hOBM)—mPCL Scaffolds

Medical‐grade polycaprolactone (mPCL, PURASORB PC12, Corbion Purac, The Netherlands) microfiber tubular (3 mm height x 4.5 mm outer diameter, 42 µm fiber diameter, 450 µm pore size) and flat (5 × 5 mm, 600 µm thickness, 40 µm fiber diameter, 250 µm pore size) scaffolds were manufactured using melt‐electrowriting technology, as per established protocols.^[^ ^9^, ^22^, ^83^ ^]^ Briefly, tubular scaffolds were made using mPCL heated to 100 °C and extruded through a 23 G tapered needle by moving an automated circular collector at a volumetric flow rate of 15 µL h^−1^, with translational and rotational speeds of 2000 mm min^−1^ and 500 mm min^−1^, respectively. Subsequently, an electrical field of 11–12 kV was applied between the needle and the collector. The scaffolds were laser‐cut to a longitudinal length of 3 mm. Flat scaffolds, used for in vitro characterization, were fabricated at a rate of 10 µL h^−1^, 20 mm above a metallic collector, and trimmed to 5 × 5 mm pieces. To confer osteoconductivity to the printed scaffolds, a calcium phosphate (CaP) coating was performed as described previously.^[^ ^84^ ^]^ Coated scaffolds were sterilized using 80% (v/v) ethanol solution for 20 min and upon evaporation, followed by UV exposition for 20 min per side before seeding the human pOBs (1.25 × 10^5^ cells per scaffold). Seeded constructs were cultured in 1 mL of GM in a humidified incubator (37 °C, 5% CO_2_) until OD and mineralization.

### In Vitro Osteogenic Differentiation and Mineralization

OD/mineralization was induced in vitro using three different biomimetic cultures (Figure 1A), for one to four weeks:

1. OM: The constructs were cultured for up to four weeks in OM (Table 2), as previously described.^[^ ^69^ ^]^
2. OD and mineralization treatment (OM+): The constructs were cultured for up to four weeks in osteogenic medium (OM, Table 2), with three days of mineralization treatment induced by mineralization medium (MM, Table 2) as previously described.^[^ ^16^ ^]^ The combination of mineralization medium with OM was chosen to enhance cell differentiation and biomineralization.
3. Mineralization treatment (GM+): The constructs were cultured for up to four weeks in basal growth medium (GM, Table 2), with three days of mineralization treatment induced by mineralization medium (MM, Table 2) as described by Thrivikraman et al.^[^ ^25^ ^]^

The human osteoblast‐derived microtissues derived from any of the treatments above are referred hereafter to as the ‘hOBMs’ and encompass both pOB‐loaded GelMA constructs and pOB‐loaded CaP‐mPCL constructs.

### In vitro cell viability pre‐implantation—Cell Metabolic Activity

Metabolic activity was measured in vitro, on the same constructs at weekly intervals using Prestoblue assay (Thermo Fisher Scientific, Waltham, MA, USA) as previously described.^[^ ^16^ ^]^ Fluorescence was measured using CLARIOStar® Plus microplate reader (BMG Labtech, Ortenberg, Germany, ex = 560 nm, em = 590 nm).

### In vitro cell viability pre‐implantation—Quant‐iT Picogreen Assay

DNA content was evaluated at day 7 and 28, following manufacturer's instructions. Briefly, the collected constructs were rinsed with PBS, transferred in tubes and frozen at ‐80°C for at least 48 h. To extract DNA, thawed samples were incubated in proteinase K solution overnight at 37 °C followed by 8 h at 56 °C. Samples were then centrifuged at 2 000 rpm for 5 min and the supernatant was used for the Picogreen assay. Standards and samples were plated in duplicates in a black 96‐well plate (100 µL per well) and incubated for 7 min with the dye solution (100 µL per well). Fluorescence was measured using CLARIOStar^®^ Plus microplate reader (BMG Labtech, Ortenberg, Germany, ex = 485 nm, em = 520nm). Calculations of DNA concentration were performed using a 4‐parameter fit analysis of the standards.

### In Vitro Osteogenic Differentiation Characterization Prior to Implantation—Gene Expression

The various hOBMs were collected after one and four weeks of in vitro culture, washed twice with PBS and incubated in TRIzol reagent (Thermofisher Scientific, Australia) at ‐80 °C for at least 48 h. After mechanically breaking constructs using a 21 G needle, RNA was isolated using Direct‐zol RNA Miniprep Plus Kit (Zymo Research, USA), following the manufacturer's instructions. Complementary DNA (cDNA) was obtained by reverse transcribing 120 ng of RNA using SensiFast cDNA Synthesis Kit (Bioline, Australia). Reverse transcriptase quantitative polymerase chain reaction (RTqPCR) was performed using the primers listed in **Table** **3**, SYBR^®^ Green PCR Master Mix (Applied Biosystems) and QuantStudio 6 Flex system (Applied Biosystems). The geometric mean of the housekeeping genes (*7SL* and *RPL32*) was used to normalize the cycle quantification (*Cq*) value of each gene, as to calculate the gene expression using the *ΔΔCq* method.

**Table 3:** Primers sequences used for RTqPCR.

Target Gene	Primers (5’‐3’)
*Collagen type 1 (Col‐1)*	F: AGGGACACAGAGGTTTCAGT R: AGCACCATCATTTCCACGAG
*Alkaline Phosphatase (ALP)*	F: ACCATTCCCACGTCTTCACATTTG R: AGACATTCTCTCGTTCACCGCC
*Osteocalcin (OCN)*	F: GATGTGGTCAGCCAACTC R: ACACTCCTCGCCCTATTG
*Osteopontin (OPN)*	F: AGACACATATGATGGCCGAGG R: GGCCTTGTATGCACCATTCAA
*Signal recognition particle RNA (7SL)*	F: ATCGGGTGTCCGCACTAAGTT R: CAGCACGGGAGTTTTGACCT
*Ribosomal protein L32 (RPL32)*	F: GCACCAGTCAGACCGATATG R: ACTGGGCAGCATGTGCTTTG

### In Vitro OD Characterization Prior to Implantation—Alkaline Phosphatase (ALP) Activity

ALP activity was measured on days 1, 7, and 28. In vitro constructs were washed with PBS 2 × 5min, transferred into 500 µL of 0.2% Triton‐1X Tris‐EDTA buffer, and stored at ‐80°C overnight. The hOBMs were repeatedly frozen (‐80°C) and thawed (37 °C) three times before being scraped using a 21 G needle. Cell lysate was collected after samples were centrifuged for 10 min at 4 °C at 10 000 rpm and mixed in para‐Nitrophenyl phosphate substrate solution (1:3 dilution, Sigma‐Aldrich). Absorbance was measured after 30 min of incubation at 405 nm. Alkaline phosphatase activity was normalized to DNA content.

### Animal Experiments

Six‐week‐old male NOD.Cg‐*Prkdc^scid^IL2rg^tm1Wjl^/SzJ* (NSG) mice were purchased from Ozgene (Perth, WA, Australia), and held at the Biological Resources Facility (Translational Research Institute, Brisbane, QLD, Australia) as approved by the University of Queensland Animal Ethics Committee (approval number 2021‐AE000353) and in accordance with the Australian Code of Practice for the Care and Use of Animals for Scientific Purposes. Mice had unrestricted access to food and water and were housed in groups of up to four mice in individually ventilated cages on a 12‐h light‐dark cycle. After one week of acclimatization, the various hOBMs were implanted subcutaneously in the mice flanks (four constructs per mouse, CaP‐mPCL scaffolds implanted below scapula, GelMA hydrogels implanted above hips) to form humanized bone tissues. Prior to implantation, the hOBMs were loaded with 22.5 µg of recombinant human bone morphogenetic protein‐2 (BMP2) (Medtronic Australasia Pty Ltd, Macquarie Park, NSW, Australia) in 40 µL of fibrin glue (TISSEEL Fibrin Sealant, Baxter Healthcare International). Mice were anaesthetized using isoflurane (4% for induction and 2% for maintenance) and Temgesic was administered subcutaneously 30 min prior to implantation (Buprenorphine, 0.05mg kg^−1^). Incisions were closed using vicryl 5‐0.16 mm sutures (Ethicon, Johnson & Johnson MedTech, New Jersey, USA). Temgesic (Buprenorphine, 0.05mg kg^−1^, subcutaneous injection) was administered every 12 h for up to 72 h post‐surgery for pain relief. In vivo mineralization was monitored weekly using microcomputed tomography (µCT) analyses (X‐Cube, Molecubes, Belgium) for 11 weeks (12 mice, n = 6 per condition). The selection of an 11‐week in vivo culture duration was based on the observed maximum in vivo mineralization formation within 7 to 11 weeks for both materials using similar methods.^[^ ^16^, ^82^ ^]^ An additional two mice were used as a negative control to assess the established need for in vivo bolus supplementation of BMP2 within hOBMs (n = 4 per condition, OM+ condition only). These animals went through the same procedures as the others. At the experimental endpoint, mice were euthanized using carbon dioxide asphyxiation, and humanized bone organs were excised and fixed with 4% (v/v) paraformaldehyde (PFA) overnight and stored in 70% (v/v) ethanol at 4 °C until further analysis.

### Mineralization

In vitro, prior to implantation:

Alizarin Red staining: Alizarin red staining was used to determine mineralization capacity of osteoprogenitors isolated from different patients, in 2D, upon OD (Figure S14, Supporting Information). Cells were fixed with ice cold methanol after two, four, and six weeks and stained with 1% alizarin red staining following the previously described protocol.^[^ ^16^ ^]^ Images were taken using phase contrast microscopy (IX73, Olympus, Australia).

Calcium assay: To determine calcium uptake from osteoblastic cells loaded on CaP‐mPCL scaffolds and GelMA hydrogels, conditioned medium was collected weekly, centrifuged for 20 min at 1000 g to remove particulates and stored at ‐20 °C. Calcium concentration from the conditioned medium was measured using a colorimetric assay adapted from a previously described arsenazo III protocol.^[^ ^85^ ^]^ Samples were plated in triplicates (2 µL undiluted conditioned medium/well) in a 96‐well plate and incubated for 5 min with 200 µL per well of assay buffer (50 mM 1,4‐ piperazinediethanesulfonic acid (PIPES, Sigma), 200 µM arsenazo III (Sigma), pH7). Absorbance at 660 nm was measured using CLARIOStar® Plus microplate reader (BMG Labtech, Ortenberg, Germany).

Microcomputed tomography: In vitro and ex vivo PFA‐fixed samples were placed in 70% (v/v) ethanol and analyzed using SkyScan 1272 µCT (Bruker, USA) to delineate the mineral deposition and measure mineralized volume (MV) and mineralized volume fraction (MV/TV). Samples were scanned using a voxel size of 12 µm, intensity of 200 µA, voltage of 50 kV and 0.25 mm aluminum filter. Samples were evaluated at a threshold of 220 to separate mineralized tissue from background noise, as previously described^[^ ^10^ ^]^ and were kept constant for every sample.

In vivo:

1) Microcomputed tomography: In vivo µCT imaging was performed using a Molecubes X‐Cube system (Molecubes, Belgium) with the following parameters: 50 kV X‐ray voltage, 350 µA current, 960 exposures at 32 ms each, continuous single rotation, and binning factor of 1. Images were reconstructed using ISRA algorithm at 50 µm isotropic voxel size, and analyzed using VivoQuant software (Invicro, Needham, MA, USA). A threshold of minimum/maximum: 300/6000 was chosen to separate mineralized tissue from background noise, as previously used.^[^ ^16^ ^]^

### Sample Resin Embedding

To characterize the ultrastructure, mechanical properties, and composition of the final bioengineered bone tissue samples, undecalcified constructs were embedded in poly(methyl methacrylate) (PMMA) resin (Technovit, Heraeus Kulzer) following the previously described protocol.^[^ ^86^ ^]^ Briefly, after dehydrating the tissues in ethanol solutions (70 to 100%) for 3 days in each solution (with two changes of 100% ethanol), samples were degreased in xylene (two changes of 4 h each). Tissues were then incubated in a Technovit 9100 pre‐infiltration solution for 4 days at 4 °C followed by 7 days in the infiltration solution. Samples were finally embedded in the low‐temperature Technovit 9100 embedding working solution and let to polymerize at ‐20 °C for 10 days. Once polymerized, the samples were sectioned (250–300 µm thick sections) using EXKAT 3100 diamond band saw mounted with a 0.1 mm blade.

### Backscattered Electron Microscopy (BSE) and Energy Dispersive Spectroscopy (EDS)

BSE and EDS were used to identify mineralized particles within the bioengineered bone tissues and determine their composition as well as the calcium/phosphate ratio (Ca/P) from their respective weight percentage (Figure S12, Supporting Information). Resin sections were grounded to 200 µm, polished using sandpapers and carbon‐coated (10 nm coating, using a Safematic carbon coater) before imaging using TESCAN MIRA3 high‐resolution electron microscope with BSE and EDS detectors using an accelerating voltage of 15.0 kV and beam intensity of 15.0 at a constant 8 mm working distance.

### Tescan Integrated Mineral Analyzer (TIMA)

Thin resin sections (100 µm) were coated with 20 nm carbon coating using Safematic carbon coater. Samples were analyzed in the Tescan Integrated Mineral Analyser (TIMA) using two EDAX Element 30 EDS detectors. Data was collected using high resolution mapping with accelerating voltage 20 kV, beam intensity 18.9 and 15‐mm working distance. A custom library was developed using the TIMA‐X software package to identify mineral phases. The field of view for individual fields was set to 100 µm.

### Histology and Immunohistochemistry

Ex vivo samples were first decalcified using 10% EDTA solution (PH 7.4) for 14 days at 37 °C using a KOS Rapid microwave lab station (ABACUS, Brisbane, Australia). Decalcified ex vivo samples were dehydrated and embedded in paraffin. Histology analyses were performed on 5 µm sections. Hematoxylin and Eosin (H&E) staining was used to characterize tissue organization and morphology using Leica Autostainer XL (Leica Biosystems, Nussloch, Germany). Immunohistochemistry (IHC) was performed to detect specific markers to characterize cellular and extracellular matrix components (**Table** **4** **),** following standard protocols.^[^ ^87^ ^]^

**Table 4:** Antibodies used for chromogenic immunohistochemistry.

Protein	Cat. No.	Company	RRID	Anti (species)	Antigen retrieval	Dilution	Incub.	Color dev.
Col‐2	II‐II6B3	DSHB (IA, USA)	AB_528165	Human, Mouse	Proteinase K, 15 min, RT	1:300	1 h, RT	1 min
Lamin A+C	ab108595	Abcam (Cambridge, UK)	AB_10866185	Human	Tris‐EDTA pH 9.0, 5 min, 95°C	1:300	1 h, RT	3 min
hCOL‐1	ab138492	Abcam (Cambridge, UK)	AB_2861258	Human	Tris‐EDTA pH 9.0, 5 min, 95°C	1:500	1 h, RT	1 min 30s
mCOL‐1	ab21286	Abcam (Cambridge, UK)	AB_446161	Mouse	Sodium Citrate pH 6.0, 5 min, 95°C	1:300	1 h, RT	4 min
OCN	ab133612	Abcam (Cambridge, UK)	AB_2916173	Human	Proteinase K, 5 min, RT	1:300	1 h, RT	3 min
OPN	ab8448	Abcam (Cambridge, UK)	AB_306566	Human, Mouse	Proteinase K, 5 min, RT	1:500	1 h, RT	3 min
DMP‐1	M176	Takara Bio (USA)	AB_2722758	Human, Mouse	Proteinase K, 5 min, RT	1:800	1 h, RT	7 min
vWf	IR52761‐2	Agilent (Dako)	AB_2810304	Human	Proteinase K, 5 min, RT	Ready‐to‐use	1 h, RT	2 min
CD68	ab125212	Abcam (Cambridge, UK)	AB_10975465	Mouse	Proteinase K, 5 min, RT	1:300	1 h, RT	2 min

TRAP staining within the decalcified ex vivo tissues was conducted following the previously detailed protocol from Van't Hof et al.^[^ ^88^ ^]^ MT and Safranin O/Fast green staining were performed to characterize collagen distribution using Leica Autostainer XL (Leica Biosystems, Nussloch, Germany). Von Kossa staining was performed on decalcified explants following the previously described protocol.^[^ ^16^ ^]^ To examine the distribution of bone matrix and calcified cartilage from calcified samples, resin thin sections (ground at 30 µm using EXKAT 400CS micro grinder) were stained with Goldner's trichrome following a previously published protocol.^[^ ^86^ ^]^

Staining examination and quantifications were performed using QuantCenter extension from Caseviewer software (3DHISTECH Ltd., version 2.4).

### Osteocyte Network and Collagen Arrangement

To visualize and quantify osteocytes and the LCN, calcified samples were stained with Rhodamine 6G (Sigma) following the previously described protocol,^[^ ^89^ ^]^ before resin embedding. Samples were imaged using fluorescence confocal laser scanning microscope (CLSM, Leica TCS SP8 DLS, Multiphoton, Leica Microsystems CMS GmbH, Wetzlar, Germany) with an oil immersion lens (ex: 520 nm, em: 550–650nm, 50ms exposure). Osteocyte density was measured using QuPath software (version 0.4.4) from stacked images (100 µm depth). Collagen fibers orientation from calcified samples was analyzed using pulsed infrared laser to detect their Second Harmonic Generation (SHG) signal (ex: 910nm, em: 450–460nm) using CLSM, and classified manually as lamellar‐like (parallels fibers) woven‐like (disorganized fibers) or intermediate using QuPath software (version 0.4.4).

Scanning electron microscopy (SEM) was used to visualize the resulting osteocyte networks from all bone tissues derived from the various hOBM types. Sections from resin‐embedded samples were cut, ground to 100 µm, and polished using sandpapers (P800, P1200, P2500 and P4000). Polished sections were then etched using 37% phosphoric acid for 3 s and 12.5% sodium hypochlorite for 5 min. After gold coating (10 nm coating using a Safematic carbon coater), sections were imaged using TESCAN MIRA3 high‐resolution electron microscope (Tescan, Brno Czech Republic) using an accelerating voltage of 5.0 kV and beam intensity of 8.0 at a constant 8 mm working distance. SEM images were used to manually quantify osteocyte alignment and the number of dendrites per osteocyte using QuPath (version 0.4.4) with Image J extension. For cell alignment quantification, cells were selected manually using ROIs selection tool and their respective angle of orientation was measured using the measured parameter “Fit ellipse” in Image J. Alignment score were determined from the variance between the measured angles from the same field of view using MATLAB software (MATLAB R2023b, The MathWorks).^[^ ^90^ ^]^

### Nanomechanical Properties

The nanomechanical properties of ossification were measured on calcified resin‐embedded polished samples using a depth‐sensing nanoindenter (Hysitron TI 980 TriboIndenter®, Hysitron Inc., USA) equipped with a standard transducer and with Berkovich diamond indenter tip. The local indentation elastic modulus (*E_r_*) and hardness (*H*) were measured by running indentation maps using eXtreme Performance Mapping (XPM) function (15 to 25 mappings per samples, grid of 9 × 9 indents separated by 5 µm in both *x* and *y* directions), with a load function of 2000 µN as the applied force. Regions of interest were selected throughout the tissues using the high‐resolution microscope attached to the transducer. Prior to indentation tests, calibration of the tip area was carefully performed on fused quartz (reduced modulus 69.9 GPa ±5% and hardness 9.25 GPa ±10%).

Elastic modulus and hardness were measured from the indentation displacement and indenter force during the loading and unloading procedure, using the Olivier‐Pharr method^[^ ^91^ ^]^:

(1) $$\mathrm{Er}=\sqrt{\pi }S/\left(2\surd \;\mathrm{Ac}\right)$$

(2) $$H=P_{\max}/A_{c}$$

where *P_max_* is the peak load, *A_c_* is the indenter contact area and *S* is the unloading contact stiffness.

2D contour maps of the local *E_r_* were generated using Origin software, with the color code representing the indentation elastic modulus (GPa). The mean *E_r_* and *H* for each region were calculated after excluding values lower than 10 GPa, corresponding to the embedding material (resin).

### Statistical Analyses and Reproducibility

All in vitro experiments were conducted on three independent biological replicates, with three to six technical replicates for each analysis. In vivo experiments were conducted on fourteen mice, with six samples per condition for implantation with BMP2, and four samples per biomaterial for implantation without BMP2. Graphs were made using GraphPad Prism version 10, showing mean ± standard deviation (SD) or mean ± standard error (SE). Statistical analyses were carried out using the General Linear Model (univariate analysis) from IBM SPSS Statistics software (version 29). Statistical significance levels were determined as *P < 0.05, **P < 0.01, ***P < 0.001 and ****P < 0.0001.

### Ethical Statement

Isolation of primary human osteoprogenitors was conducted in accordance with the ethical principles and guidelines provided by the QUT Human Research Ethics Committee (ethics approval number 1400001024). Written informed consent was obtained from all human participants involved in providing primary cells for research purposes prior to cell isolation. All experimental procedures involving animals were performed in compliance with the Australian Code of Practice for the Care and Use of Animals for Scientific Purposes, and approved by the University of Queensland Animal Ethics Committee (approval number 2021‐AE000353). Every effort was made to minimize animal suffering, and appropriate measures were taken to ensure the welfare and humane treatment of animals throughout the study.

## Conflict of Interest

D.W.H. is a co‐founder and shareholder of GELOMICS PTY LTD, a Brisbane‐based company developing and distributing hydrogels for 3D cell culture applications. All other authors declare no competing interests.

## Author Contributions

Agathe Bessot dealt with Conceptualization, Methodology, Formal analysis, Investigation, Data Curation, Visualization, Project administration, and Writing – Original Draft. Flavia Savi Medeiros carried out Methodology, Investigation, and Writing – Review & Editing. Jennifer Gunter conducted Investigation, Supervision, and Writing – Review & Editing. Jayanti Mendhidealt with Methodology, Investigation, and Writing – Review & Editing. Shahrouz Amini carried out Investigation, and Writing – Review & Editing. David Waugh conducted Funding acquisition, Supervision, and Writing – Review & Editing. Jacqui McGovern dealt with Methodology, Investigation, Supervision, and Writing – Review & Editing. Dietmar W. Hutmacher helped with Conceptualization, Methodology, Supervision, and Writing – Review & Editing. Nathalie Bock carried out Conceptualization, Methodology, Investigation, Resources, Supervision, Project administration, Funding acquisition, and Writing – Review & Editing. All authors read and reviewed the manuscript multiple times and agreed on the final curated version of the manuscript.

## Supporting information

Supporting Information

## Data Availability

The data that support the findings of this study are available from the corresponding author upon reasonable request.

## References

[1] M. A. Scott , B. Levi , A. Askarinam , A. Nguyen , T. Rackohn , K. Ting , C. Soo , A. W. James , Stem Cells Dev. 2012, 21, 655. 22085228 10.1089/scd.2011.0517 PMC3295855

[2] C. Xu , Z. Liu , X. Chen , Y. Gao , W. Wang , X. Zhuang , H. Zhang , X. Dong , Chin. Chem. Lett. 2024, 35, 109197.

[3] J. Qi , T. Yu , B. Hu , H. Wu , H. Ouyang , Int. J. Mol. Sci. 2021, 22, 10233. 34638571 10.3390/ijms221910233 PMC8508818

[4] S. Pina , V. P. Ribeiro , C. F. Marques , F. R. Maia , T. H. Silva , R. L. Reis , J. M. Oliveira , Materials 2019, 12, 1824. 31195642 10.3390/ma12111824 PMC6600968

[5] H. Qu , H. Fu , Z. Han , Y. Sun , RSC Adv. 2019, 9, 26252. 35531040 10.1039/c9ra05214c PMC9070423

[6] A. I. Alford , K. M. Kozloff , K. D. Hankenson , Int. J. Biochem. Cell Biol. 2015, 65, 20. 25997875 10.1016/j.biocel.2015.05.008

[7] P. Wang , L. Zhao , J. Liu , M. D. Weir , X. Zhou , H. H. K. Xu , Bone Res. 2014, 2, 14017. 26273526 10.1038/boneres.2014.17 PMC4472121

[8] C. Vaquette , S. Ivanovski , S. M. Hamlet , D. W. Hutmacher , Biomaterials 2013, 34, 5538. 23623428 10.1016/j.biomaterials.2013.03.088

[9] L. C. Martine , B. M. Holzapfel , J. A. McGovern , F. Wagner , V. M. Quent , P. Hesami , F. M. Wunner , C. Vaquette , E. M. De‐Juan‐Pardo , T. D. Brown , B. Nowlan , D. J. Wu , C. O. Hutmacher , D. Moi , T. Oussenko , E. Piccinini , P. W. Zandstra , R. Mazzieri , J. P. Lévesque , P. D. Dalton , A. V. Taubenberger , D. W. Hutmacher , Nat. Protoc. 2017, 12, 639. 28253234 10.1038/nprot.2017.002

[10] J. A. McGovern , N. Bock , A. Shafiee , L. C. Martine , F. Wagner , J. G. Baldwin , M. Landgraf , C. A. Lahr , C. Meinert , E. D. Williams , P. M. Pollock , J. Denham , P. J. Russell , G. P. Risbridger , J. A. Clements , D. Loessner , B. M. Holzapfel , D. W. Hutmacher , Commun. Biol. 2021, 4, 1014. 34462519 10.1038/s42003-021-02527-x PMC8405640

[11] M. Landgraf , C. A. Lahr , A. Sanchez‐Herrero , C. Meinert , A. Shokoohmand , P. M. Pollock , D. W. Hutmacher , A. Shafiee , J. A. McGovern , Bone Res. 2019, 7, 11. 31646018 10.1038/s41413-019-0072-9 PMC6804745

[12] I. Moreno‐Jiménez , A. Cipitria , A. Sánchez‐Herrero , A. F. van Tol , A. Roschger , C. A. Lahr , J. A. McGovern , D. W. Hutmacher , P. Fratzl , Sci. Adv. 2020, 6, eabb9265. 33115741 10.1126/sciadv.abb9265 PMC7608795

[13] F. Wagner , B. M. Holzapfel , J. A. McGovern , A. Shafiee , J. G. Baldwin , L. C. Martine , C. A. Lahr , F. M. Wunner , T. Friis , O. Bas , M. Boxberg , P. M. Prodinger , A. Shokoohmand , D. Moi , R. Mazzieri , D. Loessner , D. W. Hutmacher , Biomaterials 2018, 171, 230. 29705656 10.1016/j.biomaterials.2018.04.030

[14] Y. Li , Y. Liu , R. Li , H. Bai , Z. Zhu , L. Zhu , C. Zhu , Z. Che , H. Liu , J. Wang , L. Huang , Mater. Des. 2021, 210, 110049.

[15] M. Sun , X. Sun , Z. Wang , S. Guo , G. Yu , H. Yang , Polymers (Basel) 2018, 10, 1290. 30961215 10.3390/polym10111290 PMC6401825

[16] A. Bessot , J. Gunter , D. Waugh , J. A. Clements , D. W. Hutmacher , J. McGovern , N. Bock , Adv. Healthcare Mater. 2023, 12, 2201701. 10.1002/adhm.202201701 PMC11469108 36708740

[17] N. Raveendran , S. Ivanovski , C. Vaquette , Acta Biomater. 2023, 156, 190. 36155098 10.1016/j.actbio.2022.09.042

[18] H. Wang , B. Hu , H. Li , G. Feng , S. Pan , Z. Chen , B. Li , J. Song , Int J Nanomedicine 2022, 17, 1511. 35388269 10.2147/IJN.S354127 PMC8978691

[19] L. Wang , J. Mao , F. Cai , J. Tang , K. Xi , Y. Feng , Y. Xu , X. Liang , Y. Gu , L. Chen , Front Bioeng Biotechnol 2022, 10, 927050. 35935476 10.3389/fbioe.2022.927050 PMC9354842

[20] S. J. Dupard , A. Grigoryan , S. Farhat , D. L. Coutu , P. E. Bourgine , Trends Mol. Med. 2020, 26, 552. 32470383 10.1016/j.molmed.2020.01.016

[21] V. M. Quent , C. Theodoropoulos , D. W. Hutmacher , J. C. Reichert , Biomed Tech (Berl) 2016, 61, 253. 10.1515/bmt-2014-0159 25781662

[22] A. Shokoohmand , J. Ren , J. Baldwin , A. Atack , A. Shafiee , C. Theodoropoulos , M. L. Wille , P. A. Tran , L. J. Bray , D. Smith , N. Chetty , P. M. Pollock , D. W. Hutmacher , J. A. Clements , E. D. Williams , N. Bock , Biomaterials 2019, 220, 119402. 31400612 10.1016/j.biomaterials.2019.119402

[23] Y. Açil , A.‐A. Ghoniem , J. Wiltfang , M. Gierloff , J Craniomaxillofac Surg 2014, 42, 2002. 25458345 10.1016/j.jcms.2014.09.006

[24] N. S. Mahmoud , H. H. Ahmed , M. R. Mohamed , K. S. Amr , H. A. Aglan , M. A. M. Ali , M. A. Tantawy , Cytotechnology 2020, 72, 1. 31722051 10.1007/s10616-019-00353-y PMC7002803

[25] G. Thrivikraman , A. Athirasala , R. Gordon , L. Zhang , R. Bergan , D. R. Keene , J. M. Jones , H. Xie , Z. Chen , J. Tao , B. Wingender , L. Gower , J. L. Ferracane , L. E. Bertassoni , Nat. Commun. 2019, 10, 3520. 31388010 10.1038/s41467-019-11455-8 PMC6684598

[26] C. Jiani , S. Sotome , J. Wang , H. Orii , T. Uemura , K. Shinomiya , J. Med. Dent. Sci. 2005, 52, 27. 15868738

[27] F. Blaudez , S. Ivanovski , T. Fernandez , C. Vaquette , Biomacromolecules 2023, 24, 3450. 37458386 10.1021/acs.biomac.2c01504

[28] R. Finze , M. Laubach , M. Russo Serafini , U. Kneser , F. Medeiros Savi , Biomedicines 2023, 11, 2781. 37893154 10.3390/biomedicines11102781 PMC10604530

[29] I. Moreno‐Jiménez , A. Cipitria , A. Sánchez‐Herrero , A. F. van Tol , A. Roschger , C. A. Lahr , J. A. McGovern , D. W. Hutmacher , P. Fratzl , Sci. Adv. 2020, 6, eabb9265. 33115741 10.1126/sciadv.abb9265 PMC7608795

[30] P. J. Kostenuik , H. Q. Nguyen , J. McCabe , K. S. Warmington , C. Kurahara , N. Sun , C. Chen , L. Li , R. C. Cattley , G. Van , S. Scully , R. Elliott , M. Grisanti , S. Morony , H. L. Tan , F. Asuncion , X. Li , M. S. Ominsky , M. Stolina , D. Dwyer , W. C. Dougall , N. Hawkins , W. J. Boyle , W. S. Simonet , J. K. Sullivan , J. Bone Miner. Res. 2009, 24, 182. 19016581 10.1359/jbmr.081112

[31] B. M. Holzapfel , F. Wagner , D. Loessner , N. P. Holzapfel , L. Thibaudeau , R. Crawford , M. T. Ling , J. A. Clements , P. J. Russell , D. W. Hutmacher , Biomaterials 2014, 35, 4108. 24534484 10.1016/j.biomaterials.2014.01.062

[32] J. A. McGovern , A. Shafiee , F. Wagner , C. A. Lahr , M. Landgraf , C. Meinert , E. D. Williams , P. J. Russell , J. A. Clements , D. Loessner , B. M. Holzapfel , G. P. Risbridger , D. W. Hutmacher , Cancers 2018, 10, 438. 30428629 10.3390/cancers10110438 PMC6265886

[33] H. Tian , Y. Lyu , Y. G. Yang , Z. Hu , Front. Oncol. 2020, 10, 1696. 33042811 10.3389/fonc.2020.01696 PMC7518015

[34] J. Chuprin , H. Buettner , M. O. Seedhom , D. L. Greiner , J. G. Keck , F. Ishikawa , L. D. Shultz , M. A. Brehm , Nat. Rev. Clin. Oncol. 2023, 20, 192. 36635480 10.1038/s41571-022-00721-2 PMC10593256

[35] R. Ito , T. Takahashi , M. Ito , J. Cell. Physiol. 2018, 233, 3723. 28598567 10.1002/jcp.26045

[36] R. Ito , T. Takahashi , I. Katano , M. Ito , Cell Mol. Immunol. 2012, 9, 208. 22327211 10.1038/cmi.2012.2 PMC4012844

[37] B. M. Holzapfel , D. W. Hutmacher , B. Nowlan , V. Barbier , L. Thibaudeau , C. Theodoropoulos , J. D. Hooper , D. Loessner , J. A. Clements , P. J. Russell , A. R. Pettit , I. G. Winkler , J.‐P. Levesque , Biomaterials 2015, 61, 103. 26001075 10.1016/j.biomaterials.2015.04.057

[38] L. Thibaudeau , B. M. Holzapfel , D. W. Hutmacher , Cell Cycle 2015, 14, 2191. 26125650 10.1080/15384101.2015.1062327 PMC4612107

[39] L. Shen , G. Hu , C. M. Karner , Curr. Osteoporos. Rep. 2022, 20, 53. 35112289 10.1007/s11914-022-00721-2 PMC9245007

[40] S. H. Zainal Ariffin , K. W. Lim , R. Megat Abdul Wahab , Z. Zainal Ariffin , R. D. Rus Din , M. A. Shahidan , A. N. Johari , PeerJ 2022, 10, e14174. 36275474 10.7717/peerj.14174 PMC9583853

[41] C. S. Soltanoff , S. Yang , W. Chen , Y. P. Li , Crit. Rev. Eukaryot. Gene Expr. 2009, 19, 1. 19191755 10.1615/critreveukargeneexpr.v19.i1.10 PMC3392028

[42] S. Vimalraj , Gene 2020, 754, 144855. 32522695 10.1016/j.gene.2020.144855

[43] S. Boonrungsiman , E. Gentleman , R. Carzaniga , N. D. Evans , D. W. McComb , A. E. Porter , M. M. Stevens , Proc. Natl. Acad. Sci. U. S. A. 2012, 109, 14170. 22879397 10.1073/pnas.1208916109 PMC3435222

[44] N. Dirckx , M. C. Moorer , T. L. Clemens , R. C. Riddle , Nat. Rev. Endocrinol. 2019, 15, 651. 31462768 10.1038/s41574-019-0246-y PMC6958555

[45] E. Holm , J. S. Gleberzon , Y. Liao , E. S. Sørensen , F. Beier , G. K. Hunter , H. A. Goldberg , Biochem. J. 2014, 464, 355. 25310312 10.1042/BJ20140702

[46] M. Maisani , D. Pezzoli , O. Chassande , D. Mantovani , J. Tissue Eng. 2017, 8, 2041731417712073. 28634532 10.1177/2041731417712073 PMC5467968

[47] H. Cao , L. Duan , Y. Zhang , J. Cao , K. Zhang , Signal Transduct. Target. Ther. 2021, 6, 426. 34916490 10.1038/s41392-021-00830-x PMC8674418

[48] Q. Vallmajo‐Martin , C. Millan , R. Müller , F. E. Weber , M. Ehrbar , C. Ghayor , Sci. Rep. 2024, 14, 4916. 38418564 10.1038/s41598-024-55411-z PMC10901800

[49] E. Duval , C. Baugé , R. Andriamanalijaona , H. Bénateau , S. Leclercq , S. Dutoit , L. Poulain , P. Galéra , K. Boumédiene , Biomaterials 2012, 33, 6042. 22677190 10.1016/j.biomaterials.2012.04.061

[50] E. Seyedjafari , M. Soleimani , N. Ghaemi , I. Shabani , Biomacromolecules 2010, 11, 3118. 20925348 10.1021/bm1009238

[51] J. Visser , D. Gawlitta , K. E. M. Benders , S. M. H. Toma , B. Pouran , P. R. van Weeren , W. J. A. Dhert , J. Malda , Biomaterials 2015, 37, 174. 25453948 10.1016/j.biomaterials.2014.10.020

[52] P. K. Zysset , X. Edward Guo , C. Edward Hoffler , K. E. Moore , S. A. Goldstein , J. Biomech. 1999, 32, 1005. 10476838 10.1016/s0021-9290(99)00111-6

[53] S. Gesta , Y. H. Tseng , C. R. Kahn , Cell 2007, 131, 242. 17956727 10.1016/j.cell.2007.10.004

[54] O. Gurevitch , S. Slavin , A. G. Feldman , Med. Hypotheses 2007, 69, 531. 17433565 10.1016/j.mehy.2007.01.052

[55] E. Mansilla , G. H. Marín , H. Drago , F. Sturla , E. Salas , C. Gardiner , S. Bossi , R. Lamonega , A. Guzmán , A. Nuñez , M. A. Gil , G. Piccinelli , R. Ibar , C. Soratti , Transplant. Proc. 2006, 38, 967. 16647520 10.1016/j.transproceed.2006.02.053

[56] N. Alcorta‐Sevillano , I. Macías , A. Infante , C. I. Rodríguez , Cells 2020, 9, 2630. 33297501 10.3390/cells9122630 PMC7762413

[57] F. F. Safadi , M. F. Barbe , S. M. Abdelmagid , M. C. Rico , R. A. Aswad , J. Litvin , S. N. Popoff , in Bone Structure, Development and Bone Biology (Ed: J. S. Khurana ), Bone Pathology, Humana Press, Totowa, NJ 2009, pp. 1–50.

[58] Y. Ağırdil , EFORT Open Rev. 2020, 5, 498. 32953135 10.1302/2058-5241.5.190088 PMC7484711

[59] N. Alcorta‐Sevillano , I. Macías , A. Infante , C. I. Rodríguez , Cells 2020, 9.

[60] S. Von Euw , Y. Wang , G. Laurent , C. Drouet , F. Babonneau , N. Nassif , T. Azaïs , Sci. Rep. 2019, 9, 8456. 31186433 10.1038/s41598-019-44620-6 PMC6560110

[61] V. Zaichick , M. Tzaphlidou , Appl. Radiat. Isot. 2003, 58, 623. 12798370 10.1016/s0969-8043(03)00092-7

[62] W. Wang , R. Ye , W. Xie , Y. Zhang , S. An , Y. Li , Y. Zhou , Front Bioeng Biotechnol 2022, 10, 911281. 36131726 10.3389/fbioe.2022.911281 PMC9483725

[63] X. Fan , X. Wu , L. Trevisan Franca De Lima , S. Stehbens , C. Punyadeera , R. Webb , B. Hamilton , V. Ayyapann , C. McLauchlan , R. Crawford , M. Zheng , Y. Xiao , I. Prasadam , FASEB J. 2022, 36, e22142. 35032407 10.1096/fj.202101449R

[64] J. S. Nyman , M. Reyes , X. Wang , Micron 2005, 36, 566. 16169742 10.1016/j.micron.2005.07.004

[65] X. W. Su , Q. L. Feng , F. Z. Cui , X. D. Zhu , Connect. Tissue Res. 1997, 36, 271. 9512895 10.3109/03008209709160227

[66] I. L. Tsiklin , A. V. Shabunin , A. V. Kolsanov , L. T. Volova , Polymers (Basel) 2022, 14, 3222. 35956735 10.3390/polym14153222 PMC9370883

[67] M. S. Carvalho , J. M. S. Cabral , C. L. da Silva , D. Vashishth , Polymers (Basel) 2021, 13, 1095. 33808184 10.3390/polym13071095 PMC8036283

[68] Q. Leng , L. Chen , Y. Lv , Theranostics 2020, 10, 3190. 32194862 10.7150/thno.42640 PMC7053199

[69] N. Bock , A. Shokoohmand , T. Kryza , J. Röhl , J. Meijer , P. A. Tran , C. C. Nelson , J. A. Clements , D. W. Hutmacher , Bone Res. 2019, 7, 13. 31044095 10.1038/s41413-019-0049-8 PMC6486620

[70] Y. C. Chai , S. J. Roberts , E. Desmet , G. Kerckhofs , N. van Gastel , L. Geris , G. Carmeliet , J. Schrooten , F. P. Luyten , Biomaterials 2012, 33, 3127. 22269651 10.1016/j.biomaterials.2012.01.015

[71] A. Cipitria , W. Wagermaier , P. Zaslansky , H. Schell , J. C. Reichert , P. Fratzl , D. W. Hutmacher , G. N. Duda , Acta Biomater. 2015, 23, 282. 26004222 10.1016/j.actbio.2015.05.015

[72] D. H. Kempen , L. Lu , T. E. Hefferan , L. B. Creemers , A. Heijink , A. Maran , W. J. Dhert , M. J. Yaszemski , Tissue Eng Part A 2010, 16, 3769. 20666615 10.1089/ten.tea.2010.0173 PMC2991197

[73] F. E. Freeman , H. Y. Stevens , P. Owens , R. E. Guldberg , L. M. McNamara , Tissue Eng. Part A 2016, 22, 1176. 27604384 10.1089/ten.tea.2015.0339 PMC5073234

[74] F. Viti , M. Landini , A. Mezzelani , L. Petecchia , L. Milanesi , S. Scaglione , PLoS One 2016, 11, e0148173. 26828589 10.1371/journal.pone.0148173 PMC4734718

[75] Y.‐R. V. Shih , Y. Hwang , A. Phadke , H. Kang , N. S. Hwang , E. J. Caro , S. Nguyen , M. Siu , E. A. Theodorakis , N. C. Gianneschi , K. S. Vecchio , S. Chien , O. K. Lee , S. Varghese , Proc. Natl. Acad. Sci. U. S. A. 2014, 111, 990. 24395775 10.1073/pnas.1321717111 PMC3903237

[76] N. Celikkin , S. Mastrogiacomo , J. Jaroszewicz , X. F. Walboomers , W. Swieszkowski , J. Biomed. Mater. Res. A 2018, 106, 201. 28884519 10.1002/jbm.a.36226

[77] V. Karageorgiou , D. Kaplan , Biomaterials 2005, 26, 5474. 15860204 10.1016/j.biomaterials.2005.02.002

[78] G. N. Duda , S. Geissler , S. Checa , S. Tsitsilonis , A. Petersen , K. Schmidt‐Bleek , Nat. Rev. Rheumatol. 2023, 19, 78. 36624263 10.1038/s41584-022-00887-0

[79] R. Dwivedi , S. Kumar , R. Pandey , A. Mahajan , D. Nandana , D. S. Katti , D. Mehrotra , J. Oral Biol. Craniofac. Res. 2020, 10, 381. 31754598 10.1016/j.jobcr.2019.10.003 PMC6854079

[80] A. Salhotra , H. N. Shah , B. Levi , M. T. Longaker , Nat. Rev. Mol. Cell Biol. 2020, 21, 696. 32901139 10.1038/s41580-020-00279-w PMC7699981

[81] T. H. Ambrosi , T. J. Schulz , J. Mol. Med. 2017, 95, 1291. 29101431 10.1007/s00109-017-1604-7

[82] L. Thibaudeau , A. V. Taubenberger , B. M. Holzapfel , V. M. Quent , T. Fuehrmann , P. Hesami , T. D. Brown , P. D. Dalton , C. A. Power , B. G. Hollier , D. W. Hutmacher , Dis. Model Mech. 2014, 7, 299. 24713276 10.1242/dmm.014076 PMC3917251

[83] T. D. Brown , A. Slotosch , L. Thibaudeau , A. Taubenberger , D. Loessner , C. Vaquette , P. D. Dalton , D. W. Hutmacher , Biointerphases 2012, 7, 13. 22589056 10.1007/s13758-011-0013-7 PMC4875147

[84] N. Bock , Methods Mol. Biol. 2019, 2054, 23. 31482446 10.1007/978-1-4939-9769-5_2

[85] N. O. Leary , A. Pembroke , P. F. Duggan , Clin. Chem. 1992, 38, 904. 1597016

[86] D. S. Sparks , S. Saifzadeh , F. M. Savi , C. E. Dlaska , A. Berner , J. Henkel , J. C. Reichert , M. Wullschleger , J. Ren , A. Cipitria , J. A. McGovern , R. Steck , M. Wagels , M. A. Woodruff , M. A. Schuetz , D. W. Hutmacher , Nat. Protoc. 2020, 15, 877. 32060491 10.1038/s41596-019-0271-2

[87] M. Laubach , B. Herath , N. Bock , S. Suresh , S. Saifzadeh , B. L. Dargaville , J. McGovern , M.‐L. Wille , D. W. Hutmacher , F. Medeiros Savi , Frontiers in Bioengineering and Biotechnology 2023, 11, 1272348. 37860627 10.3389/fbioe.2023.1272348 PMC10584154

[88] R. J. van ’t Hof , L. Rose , E. Bassonga , A. Daroszewska , Bone 2017, 99, 69. 28366796 10.1016/j.bone.2017.03.051

[89] I. Moreno‐Jiménez , D. S. Garske , C. A. Lahr , D. W. Hutmacher , A. Cipitria , MethodsX 2021, 8, 101480. 34434878 10.1016/j.mex.2021.101480 PMC8374725

[90] F. Xu , T. Beyazoglu , E. Hefner , U. A. Gurkan , U. Demirci , Tissue Eng. Part C Methods 2011, 17, 641. 21370940 10.1089/ten.tec.2011.0038 PMC3103056

[91] W. C. Oliver , G. M. Pharr , J. Mater. Res. 1992, 7, 1564.

