# Supplementary material for: Humanized In Vivo Bone Tissue Engineering: In Vitro Preculture Conditions Control the Structural, Cellular, and Matrix Composition of Humanized Bone Organs
Source: Adv Healthc Mater. 2024 Oct 23;14(2):2401939. doi: 10.1002/adhm.202401939 (PMC11729988; doi:10.1002/adhm.202401939)
Supplement: Supplementary file 1 — Supporting Information [file ADHM-14-0-s001.docx]

**Supporting Information**

**Humanized *In Vivo* Bone Tissue Engineering: *In Vitro* Preculture Conditions Control the Structural, Cellular and Matrix Composition of Humanized Bone Organs**

*Agathe Bessot, Flavia Medeiros Savi, Jennifer Gunter, Jayanti Mendhi, Shahrouz Amini, David Waugh, Jacqui McGovern*, *Dietmar W. Hutmacher, Nathalie Bock^*^*

*Corresponding author: Nathalie Bock. Email: n.bock@qut.edu.au

**
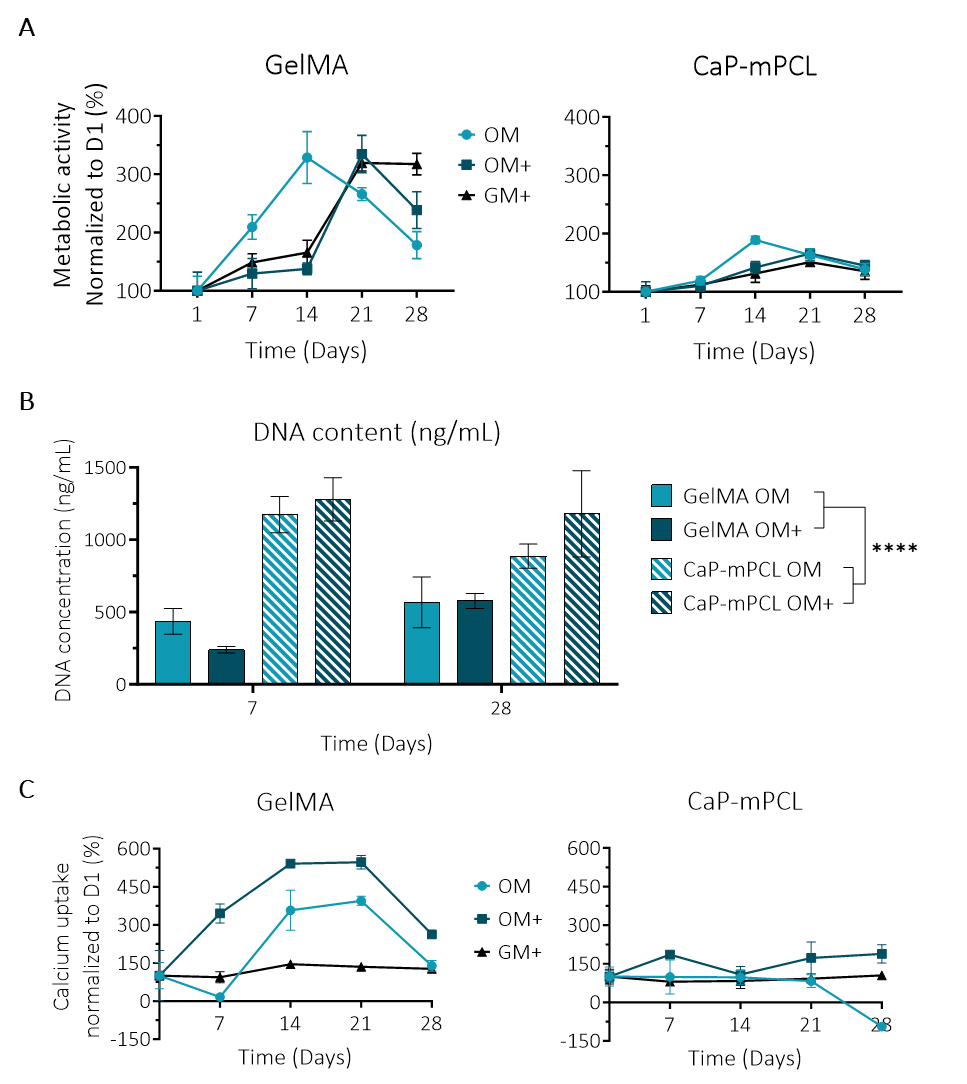
**

**Supporting Figure S1. Metabolic activity and calcium uptake characterization from *in vitro* osteoblastic microtissues. A)** Metabolic activity normalized to day one. **B)** DNA content from GelMA and CaP-mPCL constructs before *in vivo* implantation. **C)** Quantification of calcium uptake. Mean ± SD, *n* = 3, General Linear Model (Univariate), **A, C)** No significance found (all *p*>0.05 between medium conditions), **B)** *****p*<0.0001.

**
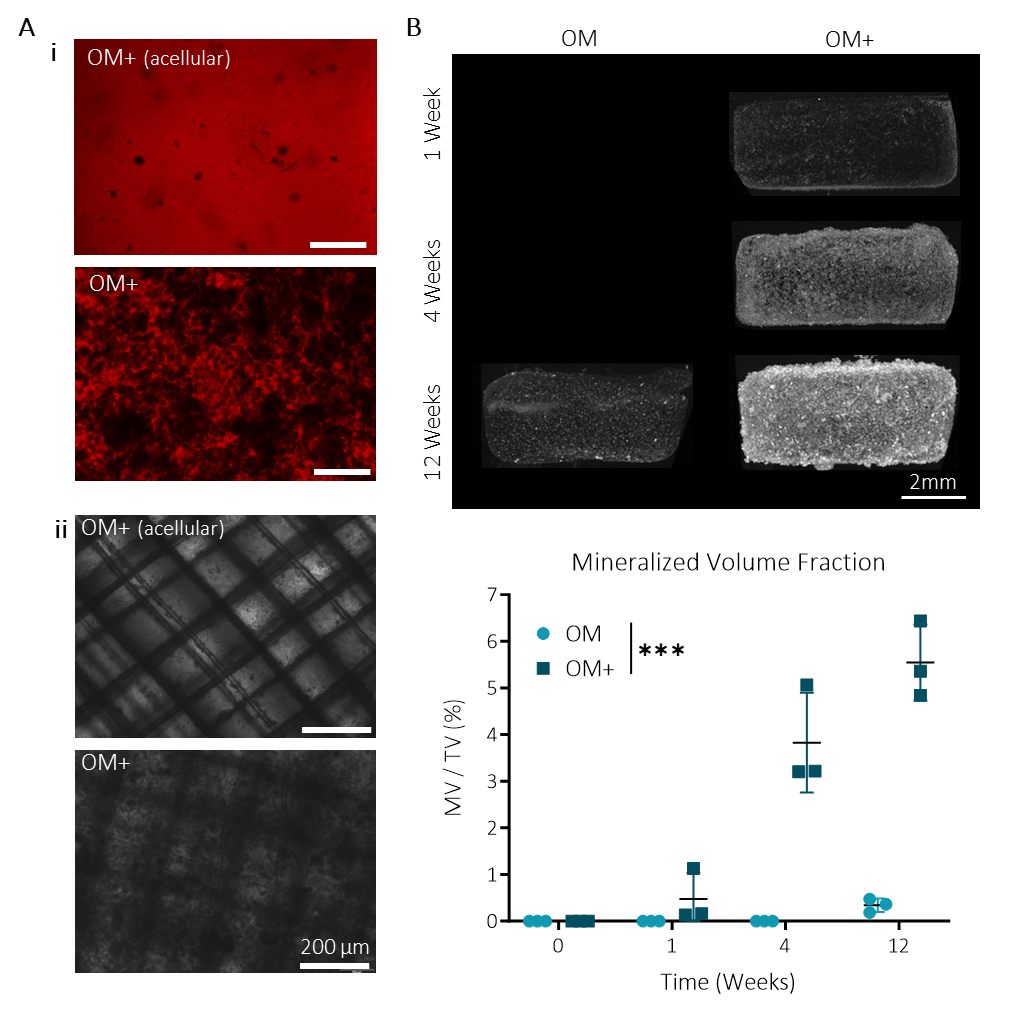
**

**Supporting Figure S2: Mineralization boost induced significantly higher mineralization compared to classic OM after up to 12 weeks of in vitro culture. A)** Alizarin Red Staining from GelMA hydrogels **(i)** and brightfield images from CaP-mPCL scaffolds **(ii)** cultured for 4 weeks in OM+ (acellular or cell-loaded constructs). Minerals in black, scale bar: 200 µm. **B)** Representative images and quantification of the mineralized volume fraction from micro-computed tomography analyses on osteoblastic GelMA hydrogels cultured in OM or OM+ for up to 12 weeks in vitro. Mean with data points, n=3, General Linear Model (Univariate), ***p<0.001.


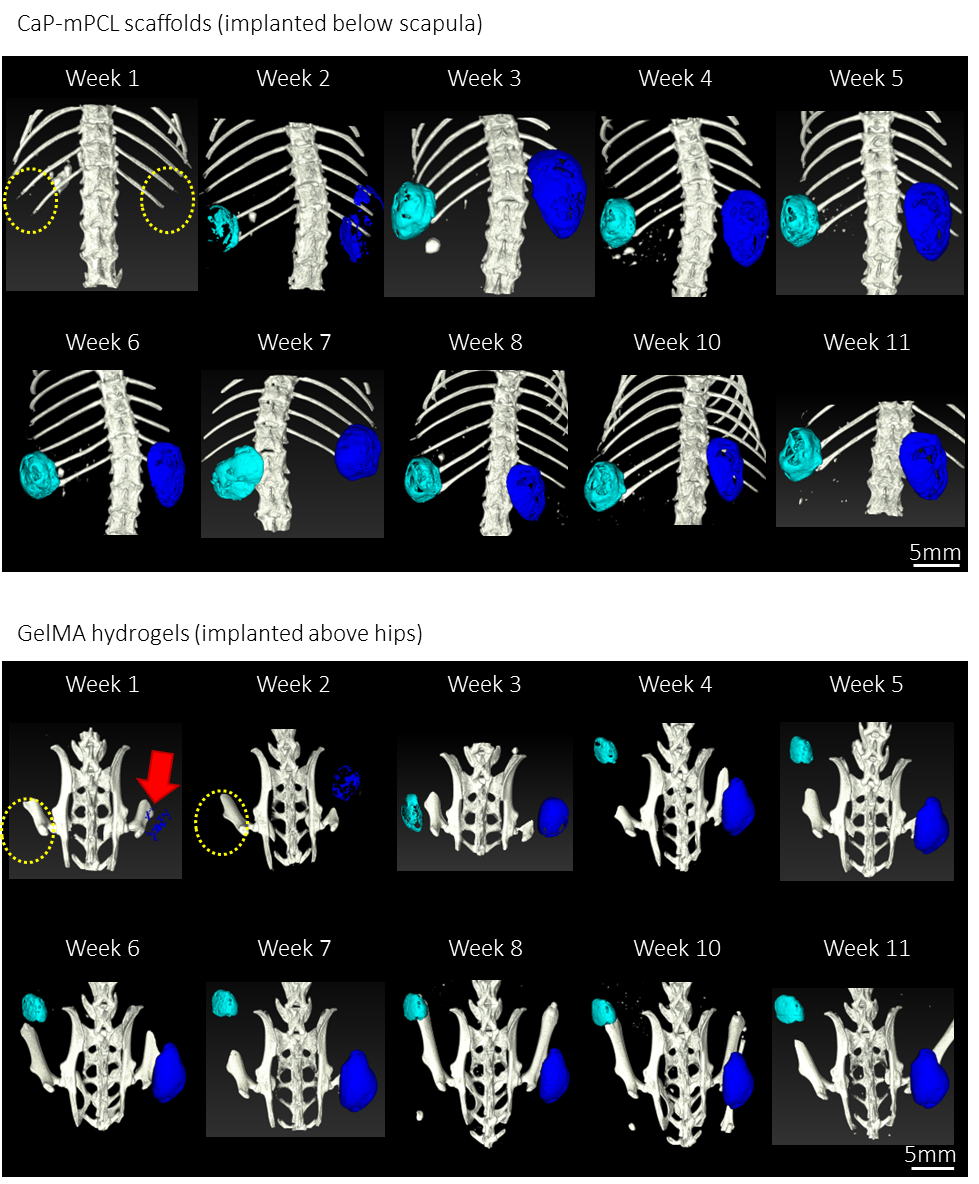


**Supporting Figure S3: Representative images of in vivo bone tissue formation overtime using µCT from 3D microtissues precultured for one week.** Representative images from in vivo µCT analyses with light blue representing OM condition and dark blue representing OM+ condition. Yellow circles showing undetectable implants.

**
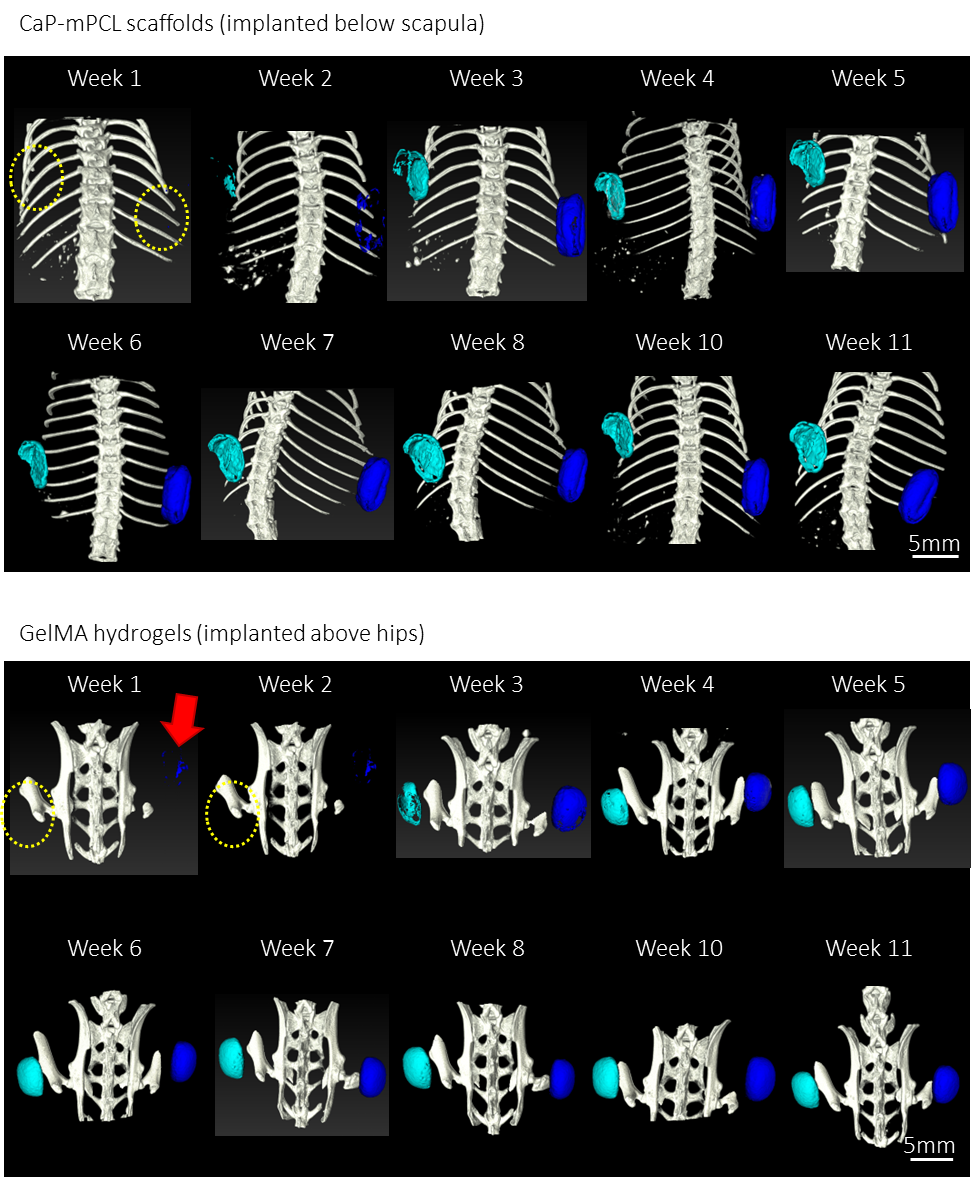
**

**Supporting Figure S4:** **Representative images of in vivo bone tissue formation overtime using µCT from 3D microtissues precultured for four weeks.** Representative images from in vivo µCT analyses with light blue representing OM condition and dark blue representing OM+ condition. Yellow circles showing undetectable implants.

**
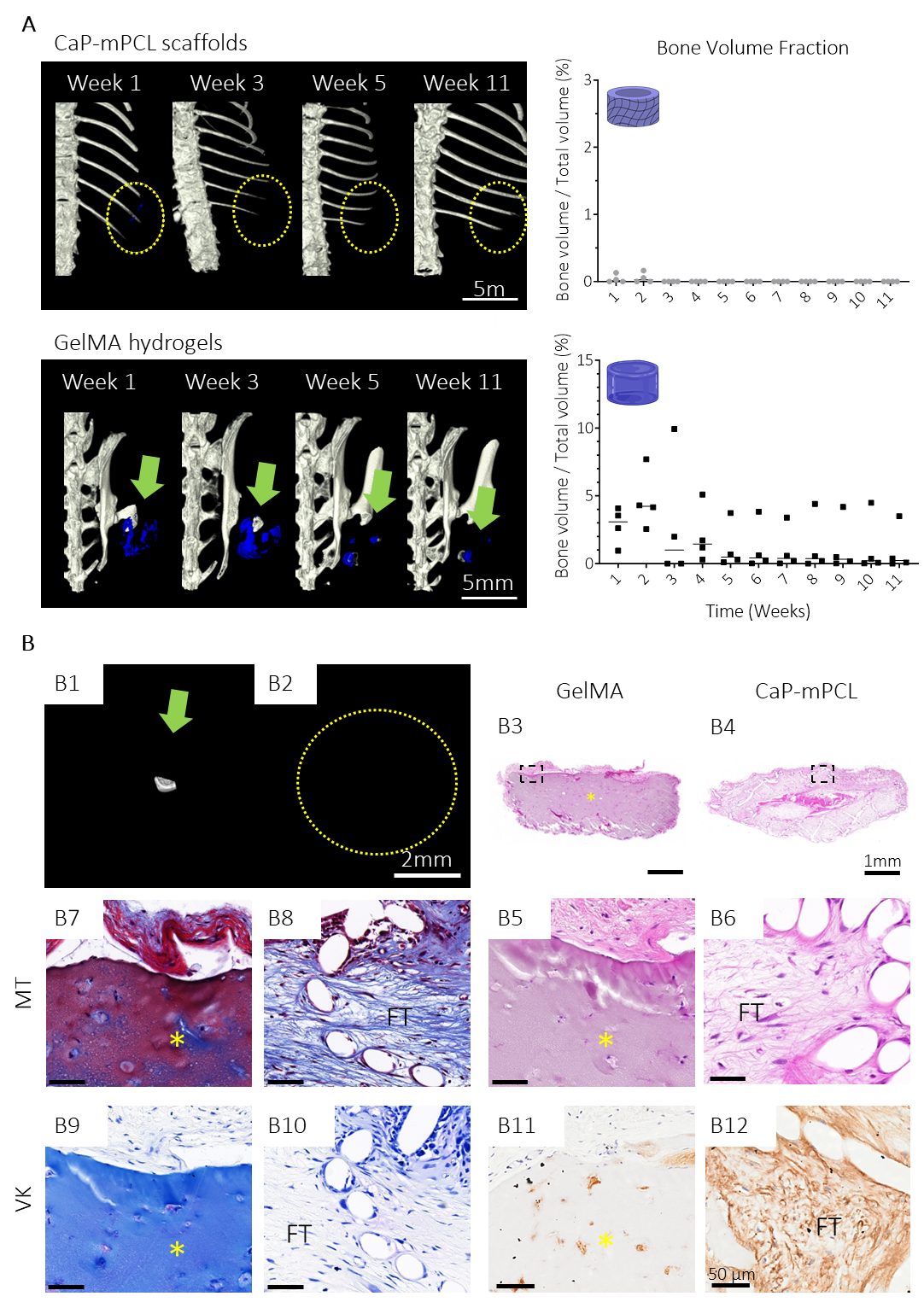
**

**Supporting Figure S5: Characterization of the explants using microcomputed tomography, histology and immunohistochemistry from 3D microtissues precultured for four weeks in OM+ condition and implanted without BMP2. A)** Representative images and quantification of *in vivo* mineralization from µCT. Yellow circles representing the location of CaP-mPCL implants (undetectable) and green arrows the GelMA implants location. **B)** *Ex vivo* characterization of constructs implanted without BMP2 with µCT (**B1-2**), and histology **(B3-6** H&E, **B7-8** Masson’s trichrome, **B9-10** Von Kossa, and **B11-12** human-specific collagen type 1 (*hCol-1*) staining) analyses. *: GelMA hydrogel, FT: Fibroblastic tissue.

**
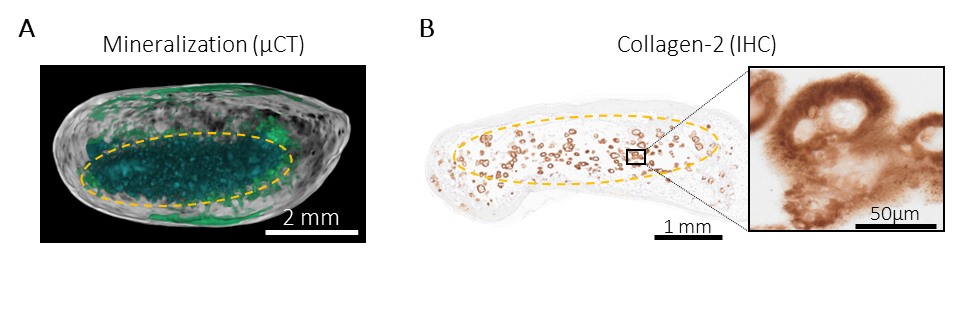
**

**Supporting Figure S6:** **Confirmation of the presence of cartilaginous tissue from GelMA hydrogel-derived explants.** Representative image of **A)** µCT and **B)** corresponding image of the *ex vivo* sample processed with IHC to detect type 2 collagen (representative images of explant derived from GelMA precultured in OM for four weeks, after 11 weeks *in vivo*). Yellow circle indicating cartilaginous tissue.


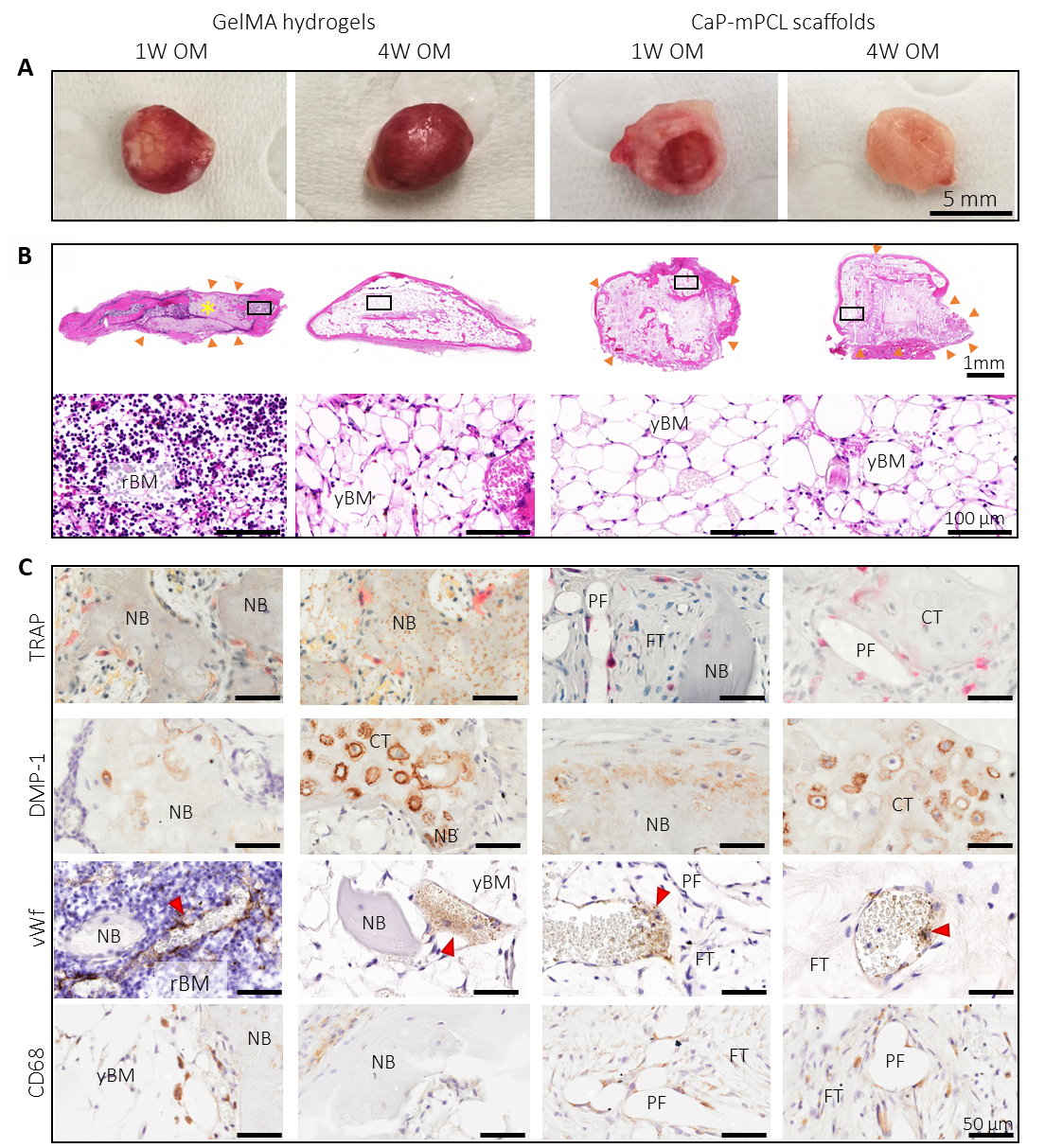


**Supporting Figure S7: Characterization of bone cellular matrix from *ex vivo* samples precultured in OM biomimetic culture. A)** Macroscopic images of collected explants after 11 weeks of culture *in vivo*. **B)** Histology analyses using H&E staining to characterize tissues morphology and marrow content, with overview (top) and high magnification (bottom). Yellow star: GelMA hydrogel, orange arrows: depleted cortical shell. **C)** IHC analyses were used to detect osteoclasts (tartrate-resistant acid phosphatase (TRAP)), osteocytes and chondrocytes (dentin matrix acidic phosphoprotein 1 (DMP-1)), vascularization (von Willebrand factor (*vWf*), red arrows) and macrophages (cluster of differentiation (CD68)) using their respective markers. NB: new bone, rBM: red bone marrow, yBM: yellow bone marrow, CT: cartilage tissue, FT: fibrous tissue, PF: CaP-mPCL fibers

**
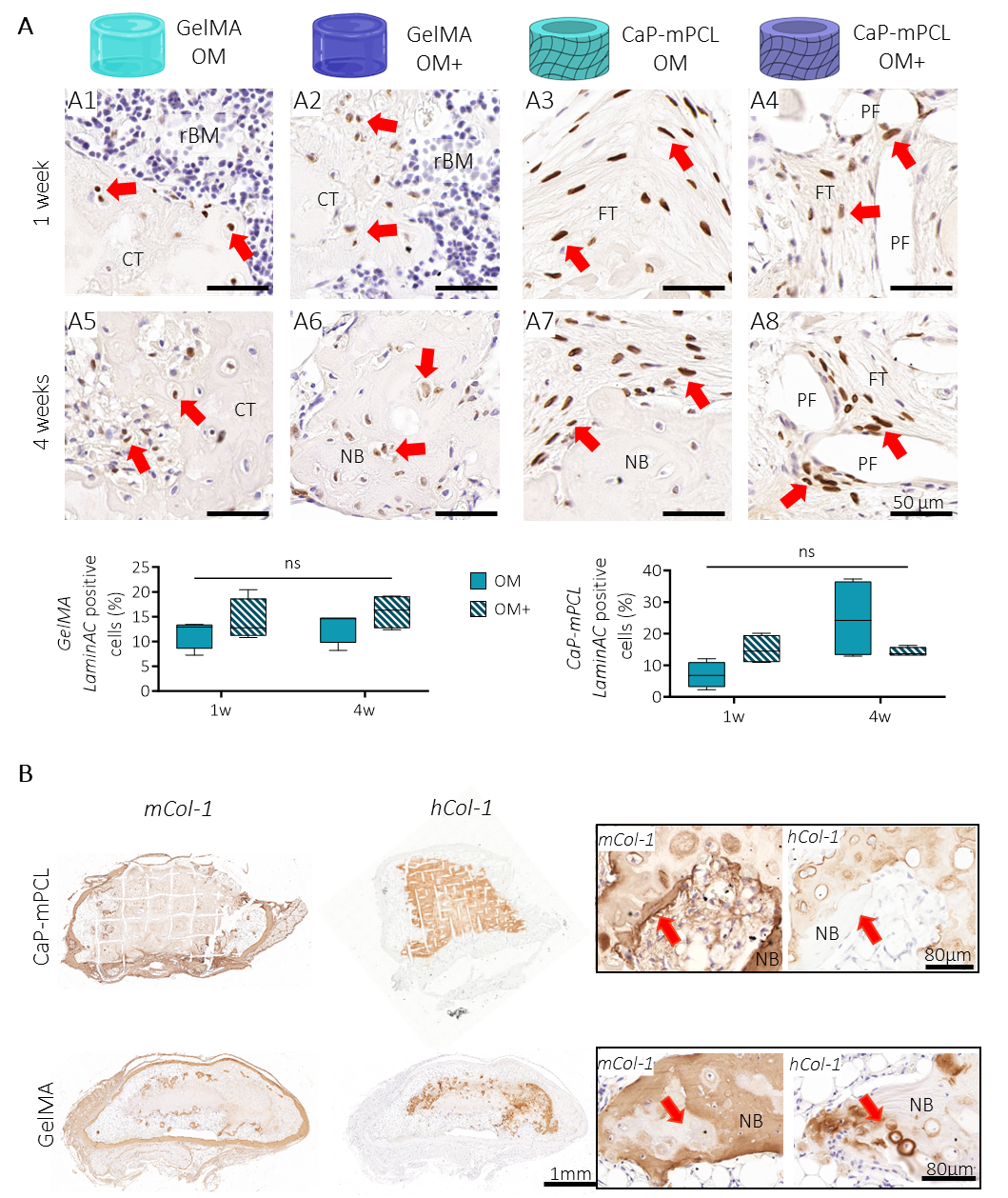
**

**Supporting Figure S8: Human cells are maintained *in vivo* and participate to the ossification process.** Histology characterization of the humanization level from the bioengineered bone samples after 11 weeks *in vivo* using **A)** Representative images of LaminAC and quantification of cells positive for LaminAC staining. Box plots with min, max and mean, *n* = 4 replicates per staining. General Linear Model  (Univariate), ns: not significant. **B)** Representative images from IHC for murine- and human-specific collagen type-1 (mCol-1 and hCol-1 respectiverly, from 4 weeks OM+ condition). Red arrows showing area positive for hCol-1. NB: new bone, rBM: red bone marrow, CT: cartilage tissue, FT: fibrous tissue, PF: CaP-mPCL fibers.

**
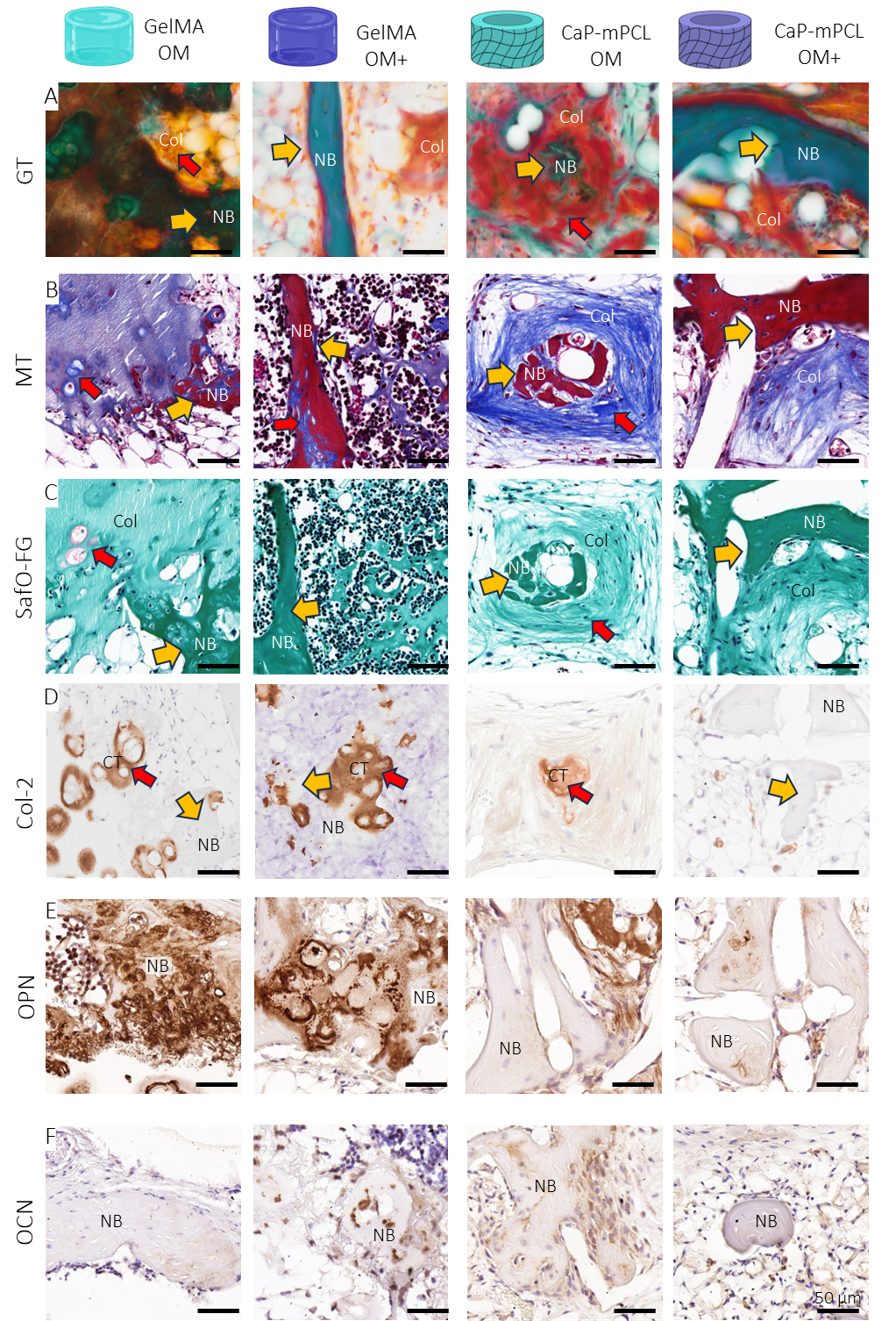
**

**Supporting Figure S9: Characterization of bone ossification and extracellular matrix from *ex vivo* samples precultured for one week.** Representative images of **A)** Goldner’s trichrome (GT), **B)** Masson’s trichrome (MT), **C)** Safranin O/Fast Green (SafO/FG) staining, **D)** collagen type 2 (Col-2) (yellow arrows: mature bone, red arrows: unmineralized collagen deposition), and the key mature bone proteins **E)** osteopontin (OPN) and **F)** osteocalcin (OCN) from GelMA and CaP-mPCL explants. NB: new bone, Col: unmineralized collagen, CT: cartilage tissue.

**
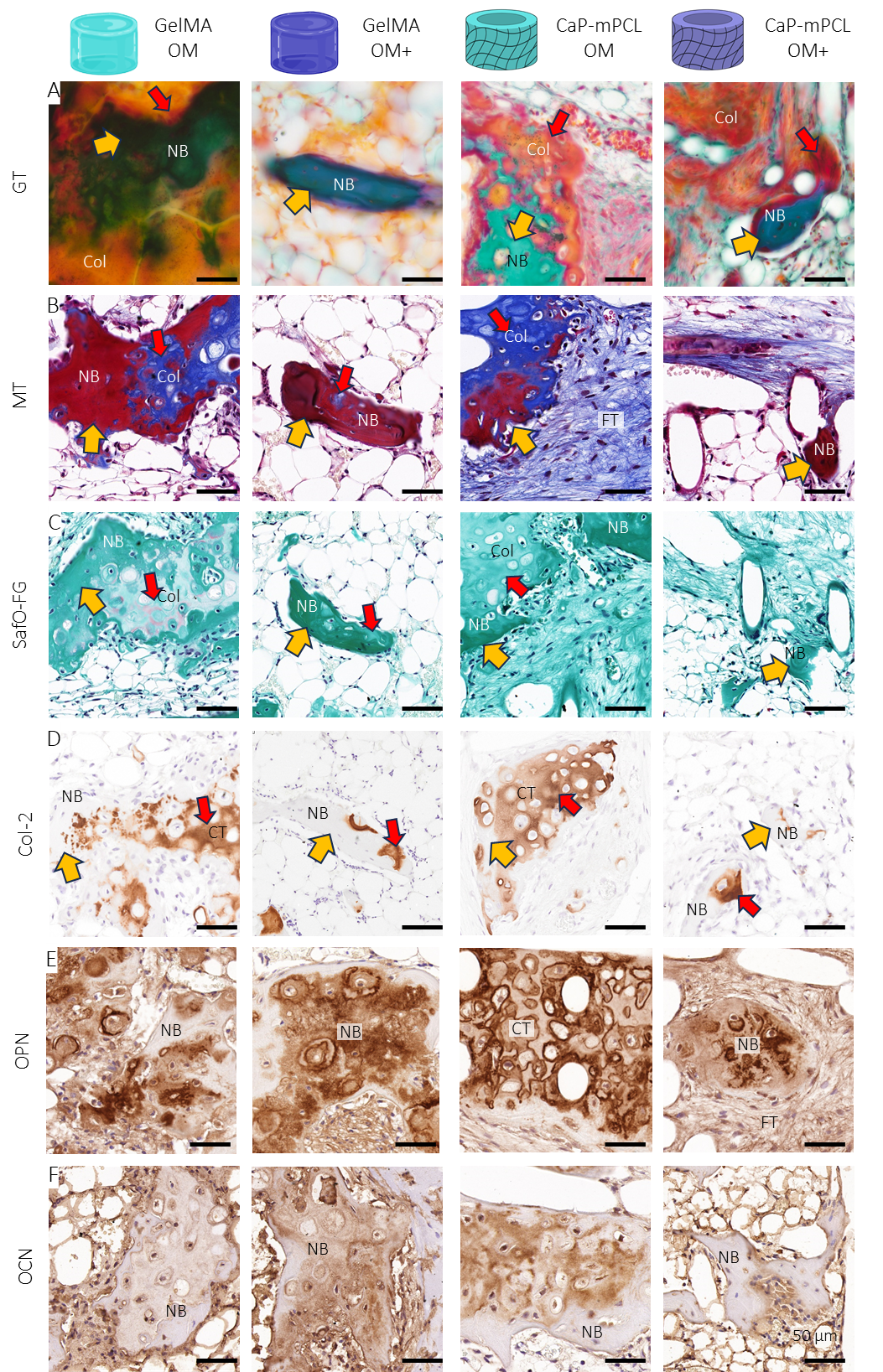
**

**Supporting Figure S10: Characterization of bone ossification and extracellular matrix from *ex vivo* samples precultured for four weeks.** Representative images of **A)** Goldner’s trichrome (GT), **B)** Masson’s trichrome (MT), **C)** Safranin O/Fast Green (SafO/FG) staining, **D)** collagen type 2 (Col-2) (yellow arrows: mature bone, red arrows: unmineralized collagen deposition), and the key mature bone proteins **E)** osteopontin (OPN) and **F)** osteocalcin (OCN) from GelMA and CaP-mPCL explants. NB: new bone, Col: unmineralized collagen, CT: cartilage tissue.


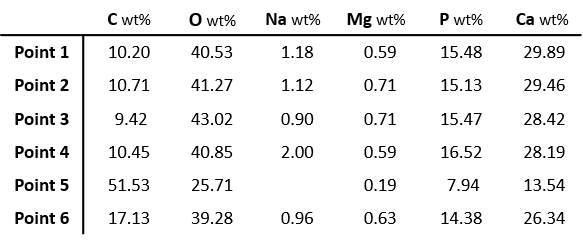


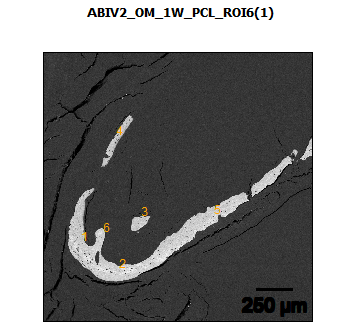


250 µm


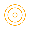

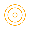

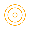

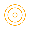

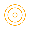


**Supporting Figure S11: Energy dispersive spectroscopy analyses using Tescan MIRA to measure Ca/P ratio.** Detection of the elements composing mineralized particles from bioengineered bone tissues using EDS, with their respective weight percentage used to calculate Ca/P ratios from region of interests.


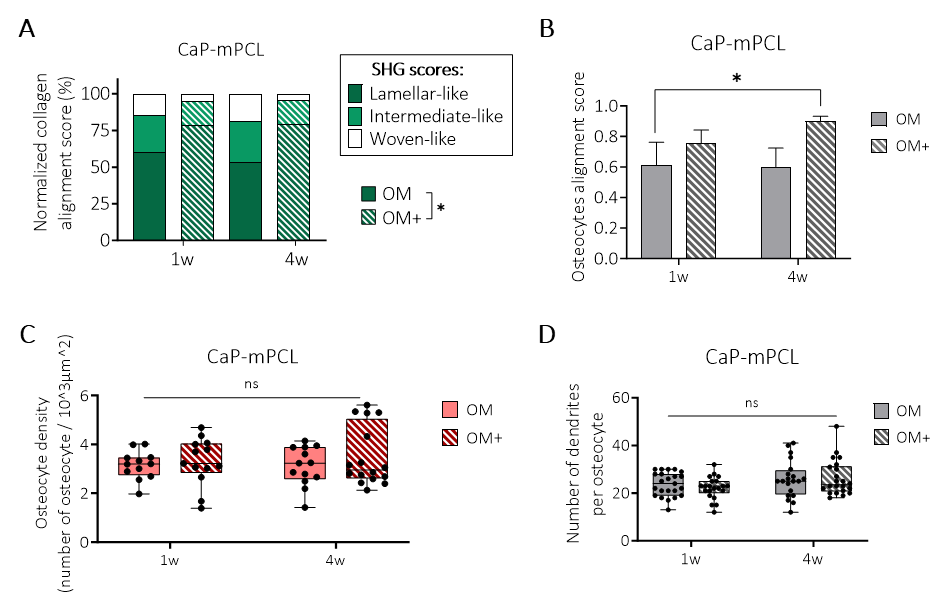


**Supporting Figure S12: Collagen fibers organization and their relationship with LCN and mechanical properties in CaP-mPCL scaffolds-derived explants**. Semi-quantification of **A)** collagen alignment score classified into lamellar-like, intermediate and woven-like arrangement (mean, *n*=3 sample per group), **B)** osteocytes alignment score (mean, *n* = 10 ROIs with a total of 190 cells analyzed per condition), **C)** osteocyte density and **D)** number of dendrites per osteocytes (box plots, min-max, *n* = 20-23 ROIs per condition). General Linear Model (Univariate), ns = not significant, **p*<0.05.


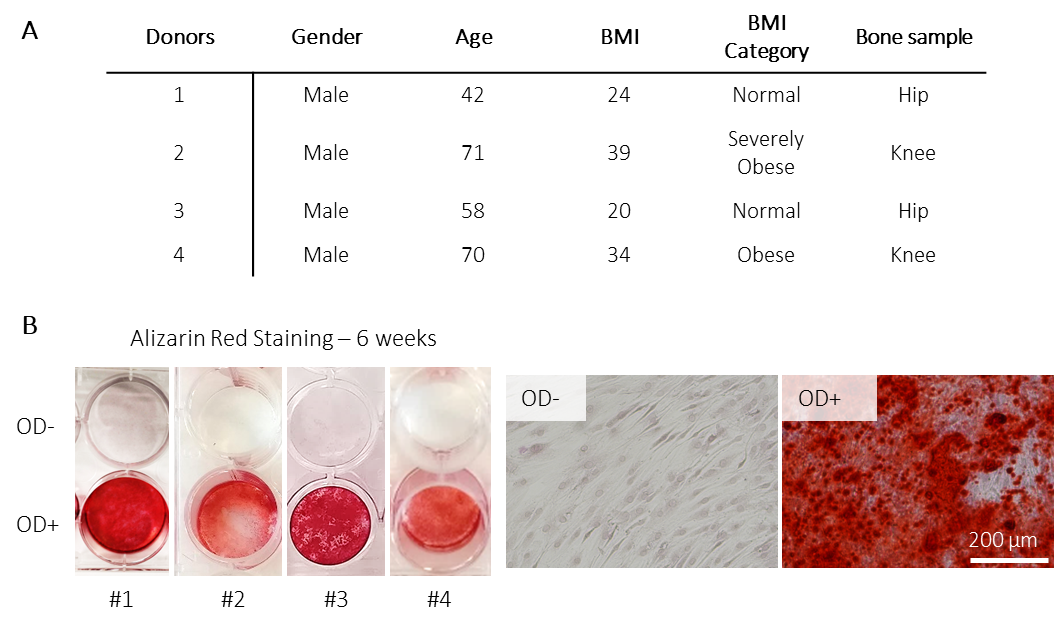


**Supporting Figure S13: Assessing mineralization capacity of primary osteoprogenitors upon osteogenic differentiation after 6 weeks of culture. A)** Table summarizing donors information **B)** Photographs and light microscopy imaging of alizarin red staining minerals deposited by primary osteoprogenitors under six weeks of culture with (OD+) or without (OD-) osteogenic differentiation in vitro (two-dimensional culture). #1-4 = Different donors, red staining = minerals, OD+ = osteogenic differentiation, OD- = no osteogenic differentiation, negative control.
